# Prevalence of lead poisoning and unique lead sources in Bhutan: Findings from a nationally representative survey 2024

**DOI:** 10.1371/journal.pgph.0006877

**Published:** 2026-07-22

**Authors:** Mongal Singh Gurung, Phillip Erbele, Kinley Dorjee, Tshering Samdrup, Sonam Wangdi, Kuenzang Dorji, Karma Lhaki, Tashi Penjor, Karma Wangdi, Krista Erbele

**Affiliations:** 1 Ministry of Health, Thimphu, Bhutan; 2 Khesar Gyalpo University of Medical Sciences of Bhutan, Thimphu, Bhutan; 3 International Partnership for Sustainable Advances in Health and Development, Lancaster, Pennsylvania, United States of America; 4 Jigme Dorji Wangchuck National Referral Hospital, Thimphu, Bhutan; University at Buffalo, UNITED STATES OF AMERICA

## Abstract

Lead is a toxic element causing devastating consequences in children and affecting health throughout life. One in three children globally are estimated to have a blood lead level (BLL) of 5 µg/dL or higher, and lead causes harm at even lower levels. Low-level lead poisoning is pervasive. However, few low- and middle-income countries have national BLL prevalence or local lead source data. A nationally representative cross-sectional BLL survey in children 1–6 years old was conducted in Bhutan in 2024. Risk factors were surveyed, and BLLs were tested with LeadCare II analyzers. Convenience sampling of children less than 13 years old in monastic institutions and purposive sampling of pregnant/breastfeeding women were conducted. Targeted environmental lead source testing was conducted using portable Evident/Olympus Vanta X-ray fluorescence (XRF) analyzers. Two BLL cutoffs were analyzed for comparison with global data. In nationally representative sampling of 1–6-year-old children, 2,246/2,959 (75.9% [95% CI 74.0-77.7]) had a BLL ≥ 3.5 µg/dL, and 1,518/2,959 (51.3% [95% CI 49.0-53.6]) had a BLL ≥ 5 µg/dL. The mean (SD) BLL was 5.3 (3.2) µg/dL. Among monastic children and pregnant/breastfeeding women, 178/207 (86.0%) and 73/124 (58.9%) respectively, had a BLL ≥ 3.5 µg/dL. These two populations were not nationally representative. Lead poisoning prevalence was high across all ages, rural/urban locations, and income quintiles. Taking *Jinlab Mendrup* (blessed religious substance) in the previous 15 days was associated with having a BLL ≥ 3.5 µg/dL, aOR=1.94 (1.34-2.70, p = 0.009). XRF source testing identified lead above reference thresholds in 44.2% (339/767) of *Jinlab Mendrup* samples, 22.4% (15/67) of spices, and 21.5% (143/665) of kitchen items. Lead poisoning prevalence was pervasive in young children in Bhutan and in other groups tested. Numerous potential lead sources were identified, including *Jinlab Mendrup* and items unique to Bhutan. Urgent action is needed to address the high prevalence of lead poisoning.

## Introduction

Lead (Pb) is a highly toxic element causing devastating consequences in all ages globally [1]. The terminology surrounding lead poisoning is at times contradictory. While the term “lead poisoning” may be applied to levels of lead toxicity requiring chelation or other clinical interventions, the term also applies to low-level lead exposure. Lead has no role in the body and causes harm at levels previously considered safe. In this study, the term ‘lead poisoning’ is used to reflect the continuum of toxic effects associated with lead exposure, consistent with evidence that no safe blood lead level exists. However, epidemiological estimates are based on established reference thresholds (≥3.5 µg/dL and ≥5 µg/dL).

Lead exposure can begin prenatally, and infants and children are especially vulnerable, as they absorb four to five times more lead than adults [1]. Absorbed lead irreversibly damages a child’s central nervous system, causing a loss in intelligence quotient (IQ), problems with learning and academics, and mental health problems at low levels, and acute encephalopathy at higher blood lead levels (BLLs) [1–5]. Low-level lead poisoning is widespread. In 2020, it was estimated that one in three children globally had a BLL of 5 micrograms per deciliter (µg/dL) or higher [1]. This is the BLL at which the World Health Organization currently recommends evaluation and mitigation of sources of lead exposure. Population data may be used to establish different levels of concern. In 2021, the CDC decreased the Blood Lead Reference Value (BLRV) for the United States to ≥3.5 µg/dL. However, there is no level of lead in the blood that is known to be safe [6–7]. IQ loss and learning problems have been shown to occur at BLLs < 3.5 µg/dL, and disproportionately more IQ loss occurs at lower compared to higher BLLs [7–9]. One fifth of the gap in learning outcomes between rich and poor countries is estimated to be due to lead [10]. Chronic low-level lead exposure is a risk factor for numerous health conditions in adults, including pregnancy complications, chronic kidney failure, and cardiovascular disease [4,11,12]. Pervasive low-level lead poisoning has health, educational, and economic consequences, which risk a country’s security [1,3]. Therefore, prevention of lead exposure is critical. Ingestion of lead represents the most common pathway of lead poisoning. However, sources and extent of lead exposure vary dramatically between countries and communities [2,13]. Lack of local data on BLL prevalence and environmental lead sources hampers effective interventions.

Bhutan is a small South Asian country striving to develop sustainably while prioritizing the concept of Gross National Happiness. With a population of fewer than one million, each person’s contribution matters in reaching the country’s development goals. However, Bhutan’s first pediatric BLL study, conducted in 2018 in the two largest cities, found 43.9% (233/531) of children 2–59 months old had a BLL ≥ 5 µg/dL, which was the level of concern at that time [14]. The study’s high prevalence was surprising, as was the lower prevalence in the industrialized city. A subsequent X-ray fluorescence (XRF) study did not identify lead sources consistent with the extensive prevalence of children’s BLLs [15]. Currently, BLL testing cannot be conducted at healthcare facilities, nor is a lead surveillance system established. Environmental testing for lead is also limited.

The objectives of the National Blood Lead Level Survey (NBLLS) 2024 were to estimate the national prevalence of children 1–6 years old in Bhutan with a BLL ≥ 3.5 µg/dL, identify associated risk factors, and determine potential sources of lead in children’s environments. This BLL level was selected to gain a fuller picture of the extent of lead poisoning. The prevalence of BLLs ≥ 5 µg/dL was also reported to facilitate comparison with global data for both cutoffs. The survey also studied the prevalence of BLLs ≥ 3.5 µg/dL among children <13 years old in monastic institutions and among pregnant and breastfeeding women. The latter two cohorts are not nationally representative samples. The data from this survey will guide interventions to decrease lead exposure and prevent lead poisoning.

## Materials and methods

### Ethics statement

Administrative clearance was obtained from the Ministry of Home Affairs and the Ministry of Health (MoH/PPD/ADM.CL/9/2024/002). The study was reviewed, and ethical clearance was granted by the Research Ethics Board of Health, Bhutan (REBH/Approval/2024/006). Enumerators explained the survey and obtained informed written consent from a parent or adult caregiver of the child, or from the pregnant/breastfeeding woman prior to the interview or blood sample collection. Verbal assent was also obtained from older children in monastic institutions, and written informed consent was obtained from the designated institutional guardian. Informed written consent was obtained from the head of household or head of school/daycare before XRF environmental testing. Interviews were conducted in Dzongkha (the national language), English, or the language most familiar to the parent or adult giving consent. Ethical standards regarding voluntary participation, confidentiality, and minimizing harm/risk were maintained.

A technical working group (TWG), led by the Research Unit, Policy and Planning Division at the Ministry of Health-Bhutan coordinated the NBLLS, 2024. Experts from IPSAHD, Khesar Gyalpo University of Medical Sciences of Bhutan (KGUMSB), UNICEF, and WHO reviewed the survey protocol and report.

### Study design, population, and sampling

The NBLLS 2024 was conducted as a nationally representative cross-sectional subset of the National Health Survey (NHS) 2023. The detailed sampling methodology is described in the NHS 2023 report [16]; key elements are summarized here.

Bhutan is administratively organized into 20 Dzongkhags (districts), which are further divided into Gewogs (blocks) and subsequently into Chiwogs (sub-blocks). For statistical purposes, the National Statistics Bureau (NSB) delineates Enumeration Areas (EAs) within Chiwogs. Urban areas are classified as towns, including four administratively independent Class A Thromde (municipalities), which are subdivided into Local Area Plans (LAPs) and corresponding EAs.

NHS 2023 employed a stratified multi-stage cluster sampling design based on the 2017 Population and Housing Census sampling frame (updated in 2022). The Primary Sampling Units (PSUs) were EAs in both rural and urban areas, with smaller Chiwogs serving as PSUs in selected remote areas. Rural and urban areas within each Dzongkhag and Thromde constituted the sampling strata, enabling estimation at national level, by rural–urban residence, and for all 20 Dzongkhags and four Class A Thromde.

In the first stage, PSUs were selected using probability proportional to size (PPS), with the number of households as the measure of size. In the second stage, 12 households per PSU were selected using circular systematic sampling. A total of 11,880 households were selected, of which 11,626 participated in the NHS 2023.

The sample size for the NBLLS 2024 was calculated in three steps. In Step 1, the sample size was estimated using the formula $n=\frac{z_{\text{1}-\alpha }^{\text{2}}p(\text{1}-p)}{d^{\text{2}}}$, where the confidence level (z) was set at 1.96 (α = 0.05). The baseline prevalence (p) was assumed to be 0.8, based on findings from Bhutan’s first blood lead level study [14], and the margin of error (d) was set at 0.05. The finite population correction was not applied, as the calculated sample size was less than 10% of the target population of children aged 1–6 years.

In Step 2, the sample size was adjusted to account for survey domains across six age groups of children and a design effect of 2. In Step 3, the sample size was further adjusted for an anticipated response rate of 80%. The final target sample size was 3,688. As the NHS 2023 included 3,627 children aged 1–6 years, all eligible children were included in the NBLLS 2024. Detailed sampling methodology for the NHS 2023 is available in the publicly accessible report (Ministry of Health, Bhutan, 2025).

All children listed in the NHS 2023 data who were ≥12 months through 6 years in March 2024 were eligible. Within the eligible households, convenience sampling of any woman who was pregnant or breastfeeding and had given birth within the previous 6 months was done. Enumerators purposively selected monastic institutions to recruit monastic children younger than 13 years old.

### Methods and variables

Local healthcare workers experienced in national surveys were selected as enumerators. Three days of comprehensive training were provided to enumerators by the TWG and faculty from the Faculty of Nursing & Public Health, including guidance on translating complex questions and on ethics related to the survey. XRF lead source training was provided to select enumerators. National data collection occurred from April 19 to June 13, 2024. Each selected child’s home was contacted and visited.

Computer-assisted personal interviews (CAPI) were used to record all data, including demographics (e.g., age, sex, woman’s education), risk factors for lead exposure at a household and individual level, and blood results. Risk factor questions included structure of the house (e.g., age, building materials), contact with lead acid batteries (e.g., solar systems, recycling), occupational risks of household members (e.g., metal artisan work, handling bullets), use of household items (e.g., cooking pots, utensils), use of traditional products (e.g., traditional medicines, kohl/eye-liner makeup), handwashing, eating practices, and the use of *Jinlab Mendrup*/blessed religious substance (e.g., if ever given, recency given).

After the interview, the enumerator collected capillary blood for BLL testing. To minimize environmental contamination from lead, lead-binding D-wipe towels (ESCA Tech, Milwaukee, WI, USA) were first used to clean a lead-free plastic work area, supplies, the tester’s gloved hands, and each participant’s hand, with particular attention to the finger selected for the blood collection. The participant’s finger was then cleaned with an alcohol wipe and pricked with a safety blade lancet. Blood was collected into a capillary tube and dispensed into a labelled LeadCare II reagent container for later analysis. A colored informational handout about lead, written in both English and Dzongkha, was provided to all participants, and prevention strategies were discussed. Finally, the caregiver was asked if they had any *Jinlab Mendrup* (blessed religious substance) which they would be willing to provide. These samples were collected for lead testing at the conclusion of the survey.

### Blood testing

The capillary BLL samples were brought to a central location daily for testing on LeadCare II analyzers (Meridian Bioscience, Cincinnati, OH, USA). The manufacturer’s collection, testing, and quality control guidelines were followed. The LeadCare II is a portable point-of-care analyzer with a BLL reportable range of 3.3-65 µg/dL. It is CLIA waived, U.S. FDA approved for screening BLLs, and its results have been verified in numerous studies [17,18]. Since BLL testing on venous blood is not available in Bhutan, and exporting samples was not feasible, any BLL ≥ 30 µg/dL was recollected and retested, and the lower of the two values was taken as the correct value. Intervention protocols were developed for any BLL ≥ 30 and ≥45 µg/dL. Enumerators called each participant or caregiver with their BLL result.

### Source testing

Selection of homes for environmental lead source testing was guided by the daily BLL results. XRF testing was offered for the home of every child with a BLL ≥ 20 µg/dL. If no result was ≥ 20 µg/dL on a day of testing, the home of the child with the highest BLL ≥ 10 µg/dL was selected. If the selected child attended an early childhood care & development (ECCD) center/daycare or school, XRF testing was conducted there as well. This strategy of source testing focused on children with the highest BLLs to identify potential lead sources in the environments of children and does not represent population-wide exposure distributions.

Evident/Olympus portable Vanta C XRF analyzers were used for environmental lead source testing. Spices, kitchen items (e.g., cookware, plates, glasses), toys, school items, soil, paint, religious and traditional Bhutanese items, and household items (e.g., doorknobs, latches, hardware) were tested with the appropriate XRF testing method. “Soil” and “RoHS” testing methods report the amount of lead in parts per million (ppm). The “Paint” method reports levels of lead in mg/cm^2^. The category of each item was recorded in a CAPI, and a picture was taken with the XRF analyzer. Samples of *Jinlab Mendrup* were requested from all households included in the survey. At the conclusion of the survey, each voluntarily provided sample was tested three times with the XRF, using the “Soil” method.

### Statistical analysis

During data collection, an independent consultant conducted periodic high-frequency quality checks. Income quintiles and rural–urban classifications from NHS 2023 were integrated into NBLLS 2024. Data cleaning, processing, and analysis were performed by the Technical Working Group (TWG).

BLL values below the LeadCare II analyzer’s limit of detection (LOD < 3.3 µg/dL) were treated as left censored. Overall, 21.5% of child BLL measurements were below the LOD. These values were imputed once using a truncated normal distribution bounded between 0 and 3.299 µg/dL and parameterised using the observed mean and standard deviation of detectable values (≥3.3 µg/dL). This bounded stochastic approach was selected over simple substitution methods (e.g., LOD/2) to better preserve distributional properties and reduce bias in estimating mean BLL while respecting biological constraints (BLL ≥ 0). Because the LOD (3.3 µg/dL) is below the primary analytical threshold (≥3.5 µg/dL), classification of children above threshold was not altered by the imputation procedure. As BLL was used to estimate population mean rather than to model causal associations, the bounded stochastic imputation approach was applied to address left-censoring and minimize bias in mean estimation. Nonetheless, sensitivity analyses were conducted using two commonly used substitution approaches for values below the limit of detection (LOD/2 and LOD/√2) to assess the robustness of mean BLL estimates.

All analyses accounted for the complex multistage sampling design. The survey design was specified in Stata using primary sampling units (PSUs), stratification (rural/urban within Dzongkhag), and sampling weights derived from the NHS 2023. The base weights were calculated as the inverse probability of selection at each sampling stage and were subsequently adjusted for household and individual non-response. The weights were normalized for analysis to maintain the effective sample size.

Finite population correction (FPC) was applied at the PSU level where appropriate. Variance estimation was conducted using Taylor series linearization. Weighted prevalence of blood lead levels (BLLs) was assessed at ≥3.5 µg/dL (US CDC BLRV) and ≥5 µg/dL (WHO intervention threshold) to facilitate comparison with globally available data [19,20]. Although no level of lead in the body is considered safe, and BLLs < 3.5 µg/dL can have adverse consequences, this survey used these two values as they represent globally accepted standards. National estimates are presented with 95% confidence intervals (CIs). Mean BLLs with standard deviations (SDs) were also reported.

Associations between explanatory variables and elevated blood lead levels were examined using survey-weighted logistic regression models. The primary outcome was defined as elevated blood lead levels (BLL ≥ 3.5 µg/dL), consistent with CDC BLRV. Because elevated BLL prevalence was relatively high, the resulting odds ratios should be interpreted as measures of association rather than prevalence ratios or risk ratios.

All models were adjusted for child age and sex, selected a priori as key demographic confounders given their established association with both lead exposure and pediatric health outcomes. Fully adjusted multivariable models were not fitted, as the objective was to estimate interpretable associations rather than causal effects. Inclusion of multiple correlated exposure variables in a single model may lead to overadjustment or difficult-to-interpret conditional associations. Each exposure variable was therefore evaluated in separate models adjusted for age and sex.

All analyses accounted for the complex survey design, incorporating clustering, stratification, and sampling weights, with variance estimation based on Taylor series linearization. Adjusted odds ratios (aORs) with 95% confidence intervals (CIs) were reported.

For variables with more than two categories, overall associations were assessed using design-adjusted Wald tests to provide a single global p-value independent of reference category selection. Category-specific inference is conveyed through the corresponding confidence intervals. Results should be interpreted as conditional associations rather than causal effects.

XRF data were exported from the analyzers to an Excel spreadsheet, merged with data from CAPI, cleaned, and analyzed. XRF data were reported as the percentages of samples having any lead above the XRF’s LOD of 2 parts per million (ppm), percentages of samples with lead levels greater than or equal to reference thresholds, and the maximum lead levels detected in each type of sample. Reference lead thresholds were chosen from international and regional standards and research studies [13,21,22]. No regulatory threshold currently exists for lead content in *Jinlab Mendrup*. Therefore, the Bangladesh standard for spices was used as the most relevant available comparator because *Jinlab Mendrup* is orally consumed and no internationally recognized lead threshold specific to this product category could be identified. [23]. Reference thresholds for paint and cosmetics were based on the US EPA and the US Center for Food Safety and Applied Nutrition’s draft recommendations [24,25].

Statistical analyses were performed using Stata/MP 18.0 (Stata Corp LLC, College Station, TX, USA).

## Results

Estimates for children aged 1–6 years are nationally representative. Findings from monastic children, pregnant or breastfeeding women, and environmental assessments are based on purposive and convenience sampling and are not nationally representative. A total of 2,959 of 3,627 eligible children 1–6 years old participated, giving a response rate of 81.6% for the nationally representative survey. Table 1 reports the number of children per age group, ranging from 411 to 581. The mean age was 49.7 (20.7) months. Female children made up 47.9% (1,415/2,959), and 39.0% (1,153/2,959) resided in urban areas. There were similar proportions of children in each income quintile. Additionally, 124 pregnant and breastfeeding women living in the households of the 1–6-year-old children were convenience sampled. Seventeen monastic institutions were visited, and 207 monastic children less than 13 years old with a mean age of 10.6 (1.8) years and primarily male, were purposively sampled.

**Table 1:** Demographic characteristics of 1-6 year old children, pregnant and breastfeeding women, and children in monastic institutions, Bhutan 2024.

Demographic	Number of participants (%)	Mean age (SD)
**Children 1–6 years old (nationally representative sampling)**		
Total	2,959 (100%)	49.7 (20.7) months
**Age (in completed years):**		
1	411 (13.9%)	18.1 (3.3) months
2	464 (15.7%)	29.6 (3.4) months
3	522 (17.6%)	41.6 (3.4) months
4	465 (15.7%)	53.7 (3.3) months
5	516 (17.4%)	65.5 (3.4) months
6	581 (19.6%)	78.1 (3.9) months
**Sex:**		
Female	1,415 (47.9%)	49.7 (20.9) months
Male	1,544 (52.2%)	49.6 (20.6) months
**Location:**		
Urban	1,153 (39.0%)	48.3 (20.6) months
Rural	1,806 (61.0%)	50.6 (20.8) months
**Income quintiles:**		
Lowest	605 (20.4%)	50.4 (20.9) months
Second	577 (19.5%)	51.4 (20.5) months
Middle	593 (20.0%)	48.0 (20.3) months
Fourth	631 (21.3%)	49.3 (21.4) months
Highest	553 (18.7%)	59.3 (20.4) months
**Pregnant and breastfeeding women in households of 1–6-year-old children (non-representative convenience sampling)**		
Total	124 (100%)	NA
**Age:**		
15–29 years	57 (46.0%)	NA
30–49 years	67 (54.0%)	NA
**Location:**		
Urban	50 (40.3%)	NA
Rural	74 (59.7%)	NA
**Education:**		
< Class 12^a^	62 (50.0%)	NA
≥ Class 12	62 (50.0%)	NA
**Children less than 13 years old from 17 monastic institutions (non-representative purposive sampling)**		
Total	207 (100%)	10.6 (1.8) years
**Sex:**		
Female	5 (2.4%)	8.8 (1.8) years
Male	202 (97.6%)	10.7 (1.8) years

^a^Includes mothers with non-formal education.

Among 2,959 children 1–6 years old, 2,246 (75.9% [CI: 74.0-77.7]), had a BLL ≥ 3.5 µg/dL and 1,518 (51.3% [CI: 49.0-53.6]) had a BLL ≥ 5 µg/dL (Table 2). Their mean BLL was 5.3 (3.2) µg/dL. A total of 635 (21.5%) observations had BLLs below the limit of detection and were imputed. Sensitivity analyses using LOD/2 and LOD/√2 substitution yielded weighted mean BLL estimates of 5.31 µg/dL (95% CI: 5.17, 5.45) and 5.46 µg/dL (95% CI: 5.32, 5.59), respectively, compared with 5.39 µg/dL (95% CI: 5.25, 5.53) using the primary truncated normal imputation approach. One-year-old children had the highest prevalence of BLLs ≥ 3.5 µg/dL and the highest mean BLL. Prevalence levels and mean BLLs trended slightly lower with age. Male children had a higher mean BLL of 5.6 (3.3) µg/dL and higher prevalences at both BLLs reported.

**Table 2:** Prevalence, confidence intervals, and mean blood lead levels by demographics of children 1-6 year, nationally representive, weighted analysis, Bhutan 2024.

Demographic Characteristics	Respondents	BLL ≥ 3.5 µg/dL		BLL ≥ 5 µg/dL		< 3.3 µg/dL^a^	Mean BLL (SD) (µg/dL)^b^
		n (%)	95% CI	n (%)	95% CI	n (%)	
Total	2,959	2,246 (75.9%)	[74.0, 77.7]	1,518 (51.3%)	[49.0, 53.6]	635 (21.5%)	5.3 (3.2)
**Age (in completed years):**							
1	411	324 (78.8%)	[74.4, 82.7]	227 (55.2%)	[49.9, 60.3]	79 (19.1%)	5.8 (3.7)
2	464	364 (78.5%)	[74.3, 82.2]	256 (55.2%)	[50.1, 60.3]	92 (19.7%)	5.6 (3.6)
3	522	386 (74.0%)	[69.5, 77.9]	263 (50.4%)	[45.6, 55.2]	127 (24.5%)	5.1 (3.0)
4	465	344 (73.9%)	[69.4, 77.9]	242 (52.1%)	[47.0, 57.2]	108 (23.1%)	5.5 (3.8)
5	516	388 (75.2%)	[70.5, 79.4]	254 (49.2%)	[44.2, 54.2]	107 (20.9%)	5.2 (3.0)
6	581	441 (75.9%)	[71.8, 79.5]	276 (47.5%)	[42.8, 52.3]	121 (21.0%)	4.9 (2.4)
**Sex:**							
Female	1,415	1,015 (71.7%)	[68.8, 74.4]	666 (47.1%)	[44.0, 50.2]	364 (25.6%)	5.1 (3.2)
Male	1,544	1,234 (79.9%)	[77.6, 82.0]	852 (55.2%)	[52.5, 57.9]	271 (17.6%)	5.6 (3.3)
**Location:**							
Urban	1,153	888 (77.0%)	[74.1, 79.7]	608 (52.7%)	[48.8, 56.6]	257 (21.6%)	5.2 (3.3)
Rural	1,806	1,358 (75.2%)	[72.7, 77.6]	910 (50.4%)	[47.6, 53.2]	378 (21.4%)	5.4 (3.2)
**Wealth quintiles:**							
Lowest	605	463 (76.5%)	[72.5, 80.1]	296 (49.0%)	[44.4, 53.6]	119 (20.5%)	5.5 (3.5)
Second	577	448 (77.6%)	[73.0, 81.6]	303 (52.5%)	[47.6, 57.5]	112 (20.1%)	5.5 (3.4)
Middle	593	440 (74.2%)	[70.1, 77.9]	299 (50.4%)	[45.8, 55.1]	140 (22.8%)	5.2 (2.9)
Fourth	631	480 (76.0%)	[71.9, 79.7]	334 (52.9%)	[48.2, 57.6]	142 (21.7%)	5.3 (3.4)
Highest	553	403 (75.6%)	[70.2, 80.3]	275 (51.6%)	[46.0, 57.2]	121 (22.0%)	5.1 (2.9)

^a^BLL below the LeadCare II analyzer’s level of detection.

^b^ BLLs below the level of detection were imputed once using a truncated normal distribution.

Among pregnant and breast-feeding women, 58.9% (73/124) had a BLL ≥ 3.5 µg/dL. Among 207 children in monastic institutions, 178 (86.0%) and 139 (67.2%) had a BLL ≥ 3.5 and ≥5 µg/dL, respectively. Their mean BLL was 5.9 (2.8) µg/dL (Table 3).

**Table 3:** Prevalence and mean blood lead levels by demographic characteristics of pregnant/breastfeeding women in households and monastic children, unweighted analysis, Bhutan 2024.

Demographic	Blood lead level, n (%)			Mean BLL (SD) (µg/dL)^b^
	≥3.5 µg/dL	≥5 µg/dL	< 3.3 µg/dL^a^	
**Pregnant and breastfeeding women in households of 1–6-year-old children (non-representative data)**				
Total	73/124 (58.9%)	37/124 (29.8%)	48/124 (38.7%)	5.2 (1.7)
**Age:**				
15–29 years	32/57 (56.1%)	16/57 (28.1%)	22/57 (38.6%)	5.2 (1.8)
30–49 years	41/67 (61.2%)	21/67 (31.3%)	26/67 (38.8%)	5.3 (1.5)
**Location:**				
Urban	28/50 (56.0%)	16/50 (32.0%)	20/50 (40.0%)	5.2 (1.4)
Rural	48/74 (60.8%)	21/74 (28.4%)	28/74 (37.8%)	5.3 (1.8)
**Education:**				
< Class 12^c^	38/62 (61.3%)	16/62 (25.8%)	23/62 (37.1%)	5.1 (1.5)
≥ Class 12	35/62 (56.5%)	21/62 (33.9%)	25/62 (40.3%)	5.4 (1.8)
**Children less than 13 years old from 17 monastic institutions (non-representative data)**				
Total	178/207 (86.0%)	139/207 (67.2%)	26/207 (12.6%)	5.9 (2.8)
**Sex:**				
Female	0/5 (0.0%)	0/5 (0.0%)	5/5 (100.0%)	1.9 (0.4)
Male	178/202 (88.1%)	139/202 (68.8%)	21/202 (10.2%)	6.0 (2.6)

^a^BLL below the LeadCare II analyzer’s level of detection.

^b^BLLs below the level of detection were imputed once using a truncated normal distribution.

^c^Includes mothers with non-formal education.

**Fig 1:**
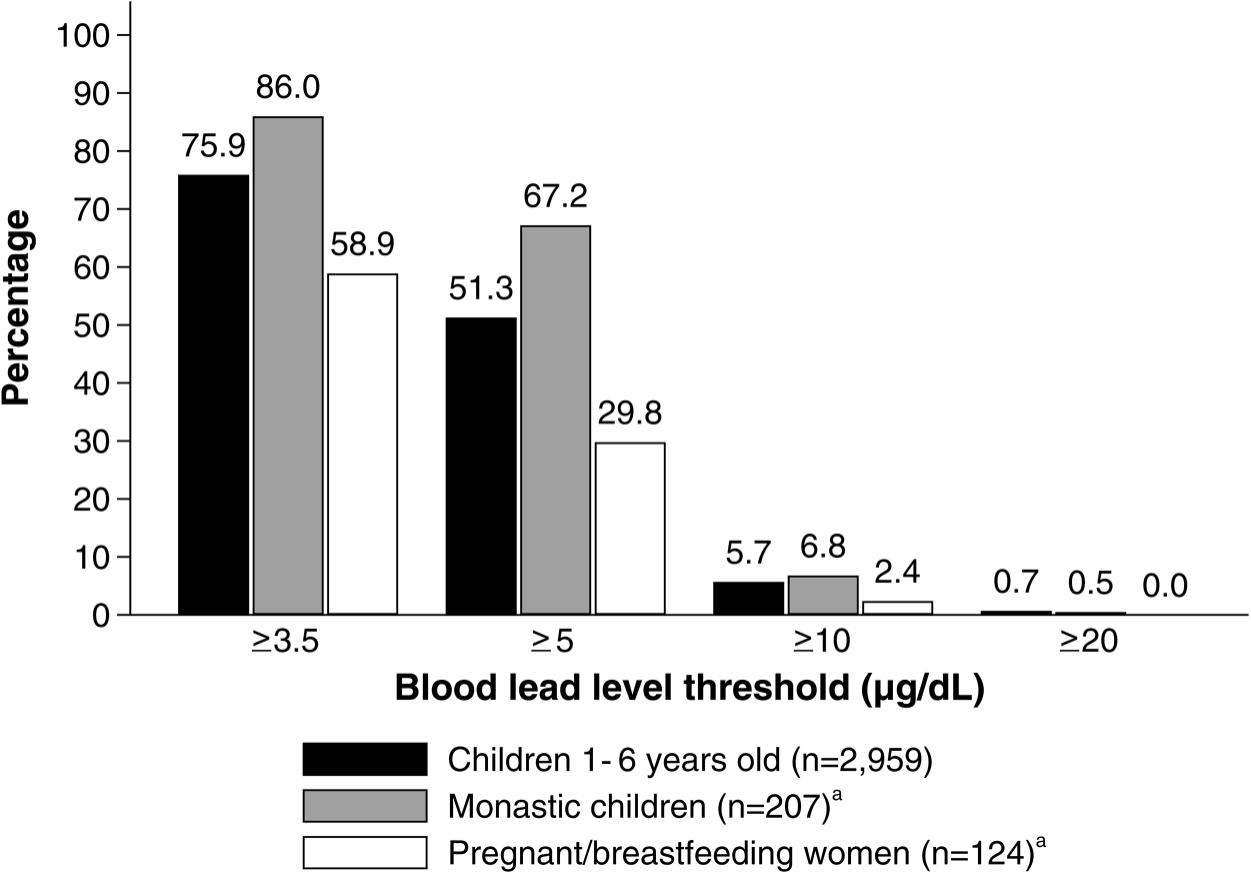
Percentage of participants with various blood lead levels. Note: ^a^ Pregnant/breastfeeding women and monastic children are not nationally representative samples.

**Fig 2:**
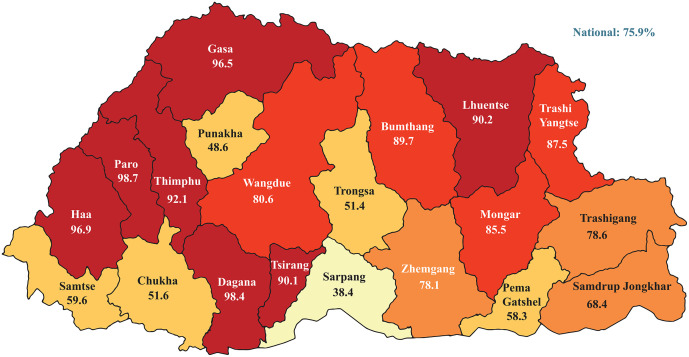
Percentages of children 1-6 years old with blood lead levels ≥3.5 µg/dL by dzongkhag (district), Bhutan.

Children from monastic institutions had higher prevalence levels at all BLLs, except at BLLs ≥ 20 µg/dL (Fig 1). The highest BLL result was 36.7 µg/dL in a child. The percentages of children with BLLs ≥ 3.5 µg/dL revealed an uneven patchwork pattern across the country. Dzongkhags (districts) in the north and northwest had higher prevalence levels compared to districts in the south (Fig 2).

Among demographic characteristics, only male sex showed a significant association (Table 4). Neither age, urban/rural location of the household, nor income showed a significant association. The age of the home did not show an association to BLLs; however, the material of the home’s interior and exterior walls showed an association to having a BLL ≥ 3.5 µg/dL, but not ≥ 5 µg/dL. Children living in homes with wood/bamboo flooring were more likely (aOR=2.42, 95% CI 1.92-3.06, p < 0.001) to have a BLL ≥ 3.5 µg/dL compared to children living in a home with a non-porous floor. The level of association was slightly less at BLLs ≥ 5 µg/dL. A household member’s use or handling of bullets showed a strong association with a child having a BLL ≥ 5 µg/dL (aOR=1.80, 95% CI 1.24-2.62, p = 0.002). Among children 1–6 years old, 66.4% (1,965/2,959) had been given *Jinlab Mendrup* in their life, and a quarter (760/2,959) had been given *Jinlab Mendrup* in the past 30 days. Having ever been given *Jinlab Mendrup*, as well as the recency of taking *Jinlab Mendrup* were both strongly associated at both BLLs. Children who took *Jinlab Mendrup* 0–15 days prior to the survey were 1.94 (aOR=1.94, 95% CI 1.34-2.80, p = 0.009) and 2.03 (aOR=2.03, 95% CI 1.51-2.72, p < 0.001) times more likely to have a BLL of ≥3.5 and ≥5 µg/dL respectively, compared to children who had never been given *Jinlab Mendrup*.

**Table 4:** Factors associated with blood lead levels ≥3.5 and ≥5 µg/dL among children 1-6 years old, adjusted Odds Ratio, adjusted for age and sex (weighted analysis) n = 2,959, Bhutan 2024.

Characteristic	Children with BLL ≥ 3.5 µg/dL			Children with BLL ≥ 5 µg/dL		
	Number (%)	Adjusted OR (95% CI)	p-value^a^	Number (%)	Adjusted OR (95% CI)	p-value^a^
**Demographic characteristics**						
**Age (in completed years)**						
1 year	324/411 (78.8%)	Ref	0.350	227/411 (55.2%)	Ref	0.096
2 years	364/464 (78.5%)	0.99 (0.71-1.38)		256/464 (55.2%)	1.01 (0.75-1.35)	
3 years	386/522 (74.0%)	0.76 (0.54-1.06)		263/522 (50.4%)	0.82 (0.62-1.08)	
4 years	344/465 (73.9%)	0.76 (0.55-1.05)		242/465 (52.1%)	0.89 (0.67-1.18)	
5 years	388/516 (75.2%)	0.81 (0.58-1.13)		254/516 (49.2%)	0.78 (0.59-1.03)	
6 years	441/581 (75.9%)	0.85 (0.62-1.16)		276/581 (47.5%)	0.74 (0.57-0.96)	
**Sex**						
Female	1,015/1,415 (71.7%)	Ref	<0.001	666/1,415 (47.1%)	Ref	<0.001
Male	1,234/1,544 (79.9%)	1.58 (1.31-1.89)		852/1,544 (55.2%)	1.39 (1.20-1.62)	
**Area**						
Urban	888/1,153 (77.0%)	Ref	0.426	608/1,153 (52.7%)	Ref	0.474
Rural	1,358/1,806 (75.2%)	0.92 (0.75-1.13)		910/1,806 (50.4%)	0.93 (0.77-1.13)	
**Income quintile**						
Lowest	463/605 (76.5%)	Ref	0.828	296/605 (49.0%)	Ref	0.743
Second	448/577 (77.6%)	1.06 (0.77-1.46)		303/577 (52.5%)	1.16 (0.89-1.50)	
Middle	440/593 (74.2%)	0.88 (0.66-1.18)		299/593 (50.4%)	1.05 (0.82-1.36)	
Fourth	480/631 (76.0%)	0.97 (0.72-1.32)		334/631 (52.9%)	1.17 (0.90-1.52)	
Highest	418/553 (75.6%)	0.96 (0.68-1.35)		285/553 (51.6%)	1.11 (0.83-1.49)	
**Housing**						
**Age of house (building)**						
< 10 years old	823/1,107 (74.3%)	Ref	0.190	549/1,107 (49.6%)	Ref	0.182
≥ 10 years old	1,426/1,852 (77.0%)	1.16 (0.93-1.44)		970/1,852 (52.4%)	1.13 (0.95-1.34)	
**Exterior wall material**						
Cement	1,240/1,680 (73.8%)	Ref	<0.001	842/1,680 (50.1%)	Ref	0.484
Rammed earth-based	758/925 (81.9%)	1.62 (1.28-2.04)		498/925 (53.8%)	1.17 (0.96-1.44)	
Wood/bamboo	222/312 (71.1%)	0.87 (0.63-1.20)		160/312 (51.3%)	1.06 (0.80-1.42)	
Other	32/42 (75.9%)	1.10 (0.48-2.52)		22/42 (51.7%)	1.09 (0.50-2.34)	
**Interior wall material**						
Cement	1,538/2,062 (74.6%)	Ref	0.027	1,039/2,062 (50.4%)	Ref	0.555
Rammed earth-based	570/709 (80.4%)	1.42 (1.11-1.82)		381/709 (53.7%)	1.16 (0.94-1.43)	
Wood/bamboo	112/152 (73.7%)	0.95 (0.58-1.55)		81/152 (53.0%)	1.11 (0.75-1.65)	
Other	30/36 (83.5%)	1.67 (0.68-4.13)		18/36 (51.2%)	1.02 (0.50-2.08)	
**Floor material**						
Concrete/tile	1,083/1,540 (70.3%)	Ref	<0.001	716/1,540 (46.5%)	Ref	<0.001
Wood/bamboo	1,005/1,185 (84.8%)	2.42 (1.92-3.06)		709/1,185 (59.8%)	1.74 (1.44-2.10)	
Mud/clay	74/90 (82.0%)	1.91 (1.06-3.47)		36/90 (39.9%)	0.76 (0.48-1.22)	
Other	91/144 (62.9%)	0.72 (0.49-1.08)		64/144 (44.1%)	0.91 (0.62-1.33)	
**Household and profession**						
**Access to car or solar system batteries** ^**b**^						
No	2,114/2,782 (76.0%)	Ref	0.423	1,422/2,782 (51.1%)	Ref	0.496
Yes, car battery	98/134 (72.8%)	0.88 (0.55-1.39)		70/134 (52.1%)	1.07 (0.72-1.59)	
Yes, solar system	33/39 (84.0%)	1.69 (0.69-4.12)		25/39 (63.6%)	1.66 (0.86-3.22)	
**A place to recycle or throw away old car batteries near house**						
No	2,196/2,890 (76.0%)	Ref	0.999	1,485/2,890 (51.4%)	Ref	0.636
Yes	52/69 (75.2%)	1.00 (0.48-2.09)		33/69 (47.9%)	0.88 (0.52-1.49)	
**Household members practice metal artisan work**						
No	2,164/2,858 (75.7%)	Ref	0.180	1,458/2,858 (51.0%)	Ref	0.150
Yes	84/101 (82.9%)	1.60 (0.81-3.17)		60/101 (59.1%)	1.41 (0.88-2.26)	
**Household members use or handle bullets**						
No	2,063/2,736 (75.4%)	Ref	0.084	1,368/2,736 (50.0%)	Ref	0.002
Yes	183/223 (82.0%)	1.46 (0.95-2.24)		144/223 (64.5%)	1.80 (1.24-2.62)	
**Kitchen items** ^**c**^						
**Aluminum cooking pots used for the preparation of food**						
No	297/403 (73.7%)	Ref	0.306	202/493 (50.0%)	Ref	0.623
Yes	1,950/2,556 (76.3%)	1.15 (0.88-1.52)		1,319/2,556 (51.6%)	1.07 (0.83-1.38)	
**Aluminum ladles/spoons used for cooking or serving food**						
No	183/267 (68.6%)	Ref	0.010	124/267 (46.5%)	Ref	0.124
Yes	2,065/2,692 (76.7%)	1.53 (1.11-2.10)		1,394/2,692 (51.8%)	1.25 (0.94-1.67)	
**Child practices**						
**Child eating with his/her fingers or hands**						
Seldom or rarely	475/619 (76.7%)	Ref	0.765	332/619 (53.6%)	Ref	0.526
Some of the time	959/1,273 (75.3%)	0.93 (0.72-1.21)		644/1,273 (50.6%)	0.88 (0.72-1.15)	
Most of the time	814/1,067 (76.3%)	1.00 (0.77-1.30)		543/1,067 (50.9%)	0.91 (0.70-1.10)	
**Hand washing before eating**						
Most of the time	1,688/2,204 (76.6%)	Ref	0.268	1,117/2,204 (50.7%)	Ref	0.712
Some of the time	506/689 (73.4%)	0.84 (0.68-1.05)		364/689 (52.9%)	1.08 (0.88-1.33)	
Seldom or rarely	53/66 (79.8%)	1.14 (0.63-2.08)		36/66 (54.5%)	1.11 (0.66-1.88)	
***Jinlab Mendrup* use**						
**Child ever given *Jinlab Mendrup* (blessed religious substance)**						
No	717/994 (72.1%)	Ref	0.001	462/994 (46.5%)	Ref	<0.001
Yes	1,533/1,965 (78.0%)	1.40 (1.14-1.72)		1,059/1,965 (53.9%)	1.37 (1.14-1.65)	
**Recency of the child being given *Jinlab Mendrup* (blessed religious substance) prior to the survey**						
Never given	717/994 (72.1%)	Ref	0.010	462/994 (46.5%)	Ref	<0.001
6 + months ago/unsure	348/444 (78.3%)	1.43 (1.05-1.94)		226/444 (51.0%)	1.23 (0.93-1.61)	
4–6 months ago	176/237 (74.3%)	1.12 (0.78-1.61)		121/237 (51.0%)	1.18 (0.87-1.61)	
1–3 months ago	397/524 (75.7%)	1.23 (0.91-1.66)		259/524 (49.4%)	1.15 (0.89-1.48)	
16–30 days ago	293/375 (78.0%)	1.41 (1.00-2.00)		208/375 (55.5%)	1.48 (1.10-1.99)	
0–15 days ago	320/385 (83.1%)	1.94 (1.34-2.80)		244/385 (63.3%)	2.03 (1.51-2.72)	

^a^Adjusted Wald test.

^b^Four households had both types of batteries and all four had an elevated BLL.

^c^The child may or may not have used these kitchen items. The information was about household use of these items.

Environmental testing using XRF was conducted in purposively selected high-risk households and institutions and therefore does not represent population-level prevalence of lead-containing sources. Results from environmental source testing with the XRF found multiple items containing lead, representing potential exposure pathways. Of the 767 samples of *Jinlab Mendrup* collected during the survey, 339 (44.2%) had ≥ 2.5 parts per million (ppm) of lead, and the maximum level detected was 57,233 ppm (Table 5). Source testing was conducted in 67 homes, 6 ECCDs, and 4 schools. Of 67 spices tested, 15 (22.4%) contained ≥2.5 ppm of lead; the maximum lead level detected was 202 ppm. One in five kitchen items tested had ≥ 100 ppm of lead. Nineteen toys tested contained ≥100 ppm of lead; the maximum level detected was 56,122 ppm. Only one out of 127 paint samples tested contained lead above 1 mg/cm^2^. Three quarters of religious and traditional items unique to Bhutan contained lead above the XRF’s level of detection of 2 ppm.

**Table 5:** Samples tested by portable X-ray fluorescent analyzer and percentage of items exceeding thresholds.

Items tested	Lead reference threshold^a^	Number tested	Number (%) with any lead (>LOD)^b^	Number (%) with lead ≥ reference threshold	Maximum lead level>LOD
*Jinlab Mendrup* (blessed religious substance)	2.5 ppm	767	340 (44.3%)	339 (44.2%)	57,233 ppm
Spices	2.5 ppm	67	28 (41.8%)	15 (22.4%)	202 ppm
Kitchen, all items	100 ppm	665	300 (45.1%)	143 (21.5%)	38,190 ppm
Cosmetics	10 ppm	14	2 (14.3%)	2 (14.3%)	25 ppm
Toys	100 ppm	209	48 (23.0%)	19 (9.1%)	56,122 ppm
School items	100 ppm	41	6 (14.6%)	4 (9.8%)	3,300 ppm
Soil	200 ppm	60	49 (81.7%)	2 (3.3%)	502 ppm
Paint	1 mg/cm^2^	127	38 (29.9%)	1 (0.8%)	1 mg/cm^2^
Religious & traditional Bhutanese items	NA	214	161 (75.2%)	NA	788,537 ppm
Household items	NA	246	116 (47.2%)	NA	144,086 ppm
Total		2,410			

^a^Reference thresholds are based on US and Bangladesh thresholds and on research. No thresholds exist for some categories.

^b^LOD – level of detection for lead using portable X-ray fluorescence is 2 ppm.

## Discussion

The NBLLS 2024, the first national survey of blood lead levels in Bhutan, found pervasive low-level lead poisoning across all groups tested. Among 1–6-year-old children tested, 75.9% (2,246/2,959) had a BLL ≥ 3.5 µg/dL, and 51.3% (1,518) had a BLL ≥ 5 µg/dL. Convenience sampling of pregnant and breastfeeding women found 58.9% (73/124) with BLLs ≥ 3.5 µg/dL. Children under age 13 in monastic institutions, sampled purposively, had the highest mean BLL and the highest BLL prevalence, with 86.0% (178/207) having a BLL ≥ 3.5 µg/dL. These findings seem surprising in a country with limited industrialization. However, they corroborate the 2018 study done in two of Bhutan’s cities in which 50.7% (216/426) of children 1–4 years old had a BLL ≥ 5 µg/dL [14]. Several local sources of lead exposure were identified, highlighting the need for LMICs to have local data. XRF source testing identified lead in 44.2% (339/767) of *Jinlab Mendrup* samples (blessed religious substance), 22.4% (15/67) of spices, and 21.5% (143/665) of kitchenware. This study provides the first evidence identifying *Jinlab Mendrup* as a potential source of lead exposure. Although causality cannot be established from this cross-sectional study, the observed associations, biological plausibility, and detection of lead in *Jinlab Mendrup* samples support further investigation and appropriate preventive measures in Bhutan and other settings where similar products are used.

Comparison of Bhutan’s findings with other LMICs is limited by lack of nationally representative data. The prevalence of blood lead levels in LMICs is largely based on estimates [2,26]. Several countries report lower national prevalence levels of children with a BLL ≥ 5 µg/dL compared to our study: the Republic of Georgia (41%), Mexico (16.8%), and China (2.7%) [2,27,28]. The US reports that 2.5% of children had a BLL ≥ 3.5 µg/dL, and in France, 2.9% of children had a BLL ≥ 2.5 µg/dL [19,29]. Regional BLL data is comparable to our findings or even higher. However, most BLL data from neighboring India, Nepal, and Bangladesh are from smaller studies, often focusing on hotspots [25,30–33]. A representative study of 697 children in the state of Bihar, India found an alarming 89.7% of children had a BLL ≥ 5 µg/dL [25]. The mean BLL among 1–6-year-old children in our survey was 5.3 (3.2) µg/dL. This is dramatically higher than nationally representive mean BLLs from China, US, and EU countries [27–29,34–36]. However, studies focusing on areas of high lead exposure in Bihar, India and Nepal have shown mean BLLs of 8.2 and 20.3 µg/dL, respectively [25,37].

Although it was not nationally representative data, the children in monastic institutions had the highest prevalence at both ≥3.5 and ≥5 µg/dL BLLs. Their mean BLL was 5.9 (2.8) µg/dL, 10% higher than the mean BLL in 1–6-year-old children. As these children were on average 6 years older, this suggests that monastic children may have higher exposures to lead compared to non-monastic children. Comparison lead studies focused on monastic communities are very limited. In one study among Hinayana Buddhist monks in Thailand, a high prevalence and mean BLL of 38.2 µg/dL was found [38]. Further studies in monastic populations are needed.

Lead poisoning has a generational impact. As lead can cross the placenta to the developing fetus, the prevalence rates in pregnant/breastfeeding women confirm that many children in Bhutan may be exposed to lead even before birth. This data was collected conveniently from women in homes of children in the survey. While it is not nationally representative, it indicates the need for further research. For comparison, a study in Bihar state identified an alarming 81.8% of pregnant women had a BLL ≥ 5 µg/dL, with a mean BLL of 9.0 µg/dL [25]. Blood lead levels in a pregnant woman result both from ongoing exposure and from release into the blood stream from lead sequestered in bones decades earlier. Interventions to reduce lead during childhood will reduce lead exposure for future pregnancies.

Several significant associations were found in the survey. As in nearly all studies, males had a significantly higher prevalence at both BLLs reported. Homes with wood/bamboo flooring showed a high association with BLLs. This is possibly from the collection of lead dust between the boards. Access to a lead acid battery or a battery recycling location was not associated with BLLs in contrast to other studies [2,25]. In Bhutan’s first BLL study, eating with ones’ hands was associated with BLLs [14]. This survey, however, did not show a similar finding.

One notable association was observed with the use of *Jinlab Mendrup*, a blessed religious substance traditionally prepared from hundreds of ingredients, including healing herbs and medicinal components. Although not registered as a traditional medicine, it is commonly consumed for therapeutic purposes and is distributed by religious figures rather than traditional medicine practitioners. *Jinlab Mendrup* is also called *jinlab*/*mendrup*/*damzey*/*rilbu*, and is made in diverse shapes, sizes, and colors. Both ever having received *Jinlab Mendrup* and the recency of its consumption were strongly associated with blood lead levels. Our survey found 66.4% (1,965/2,959) of children had been given *Jinlab Mendrup* in their life. Although this does not show causation, these factors align with the highly significant associations between taking *Jinlab Mendrup* and BLLs in children. Socio-economic, cultural, and religious practices could represent additional confounding factors. To our knowledge, no study has reported on the association of *Jinlab Mendrup* use and BLLs in children. Case reports on lead poisoning have been reported from Chinese and Tibetan medicine [39–41]. Ancient documents on the use of Tibetan medicine and *Jinlab Mendrup* report side effects such as vomiting, diarrhea, and abdominal pain, which are consistent with lead poisoning [42].

Testing *Jinlab Mendrup* for lead was part of the environmental source testing. Because it is consumed, any lead present in *Jinlab Mendrup* would contribute directly to lead poisoning. At each home visited during the survey, a sample of *Jinlab Mendrup* was requested for testing at the end of the survey. XRF testing found lead in 44.2% (339/767) of *Jinlab Mendrup* samples provided to enumerators. Thus, it is a potential risk for anyone who takes it. *Jinlab Mendrup* is traditionally taken early in the morning on an empty stomach, a practice which would increase the absorption of any lead. Because *Jinlab Mendrup* is used across Himalayan and Tibetan-Buddhist communities globally—including parts of India, Nepal, Tibet, Mongolia, and worldwide diaspora communities—this cultural exposure pathway likely extends beyond Bhutan and merits coordinated regional and diaspora-focused further studies and risk mitigation.

It is notable that *Jinlab Mendrup* is only one of numerous potential sources of lead identified in XRF testing. As 72.1% (717/994) of children who reported never being given *Jinlab Mendrup* had a BLL ≥ 3.5 µg/dL, other sources of lead are present. Further source testing is needed to establish clear links between sources of exposure and blood lead levels.

Ingestion of lead represents a common pathway of lead poisoning. Items identified by the XRF as containing lead could pose an ingestion risk, for example by leaching into food or via contaminated hands. XRF source testing at children’s homes with the highest BLLs identified kitchen items and toys among the household items containing lead. This reflects the widespread presence of lead in manufactured consumer goods in the region and is consistent with the findings of the rapid market screening report conducted by Pure Earth in 25 LMICs [13]. This also highlights the need for regulation and enforcement in both local and regional manufacturing of consumer goods.

Lead was also identified in spices, although to a lesser extent compared to Bangladesh, India and the Republic of Georgia [13,43,44]. Many religious and Bhutanese items, for which there are no reference thresholds, were found to have lead. Although the contribution of these items to blood lead levels could not be directly quantified, promoting handwashing after handling such items may represent a simple and culturally acceptable precautionary measure while further studies investigate potential exposure pathways. Additional research is needed to follow up the XRF findings and search for additional sources.

This survey identified the presence of lead in many items within the environments of children in Bhutan. This knowledge can guide Bhutan’s government and development partners to urgently strengthen laboratory and human capacity to address this issue. Additionally legal regulatory guidelines can be developed to limit sources and regulate lead.

Limitations of the survey include the use of the LeadCare II analyzer, which has a LOD of 3.3 µg/dL and potential contamination risks associated with capillary blood sampling. Although rigorous quality control procedures including using D-wipe towels (ESCA Tech) were implemented, these limitations should be considered when interpreting the findings. Confirmatory venous testing was not possible. Bhutan does not have a laboratory for blood lead testing, and the logistics, costs, and ethical concerns of drawing a venous sample for laboratory confirmation outweighed the benefits. Also, source testing was only done at homes of children with the highest BLLs, and the testing was limited to portable XRF testing. The enumerators conducting the XRF testing had limited training and experience and may have overlooked potential lead sources. Laboratory testing capacity to conduct extensive water, air, leach testing, and testing to parts per billion was not available in country at the time of the survey. Although a bounded imputation approach was used to preserve distributional properties, any imputation of left-censored values may introduce uncertainty in estimates of mean BLL. Given the cross-sectional design, associations observed cannot establish temporal direction or causality.

## Conclusion

The widespread prevalence of lead poisoning in Bhutan has implications for the nation’s social, health, and economic future. There is no level of lead in the body that is known to be safe. Even low levels of lead are known to cause permanent consequences. The development of laboratory testing capabilities and further research in at-risk populations are needed. These findings require an urgent multi-sectoral approach to identify and address all sources of lead.
