# Supplementary material for: Prevalence of lead poisoning and unique lead sources in Bhutan: Findings from a nationally representative survey 2024
Source: PLOS Glob Public Health. 2026 Jul 22;6(7):e0006877. doi: 10.1371/journal.pgph.0006877 (PMC13390818; doi:10.1371/journal.pgph.0006877)
Supplement: Attachment — Submitted filename: Response to Reviewers.docx [file pgph.0006877.s002.docx]

Manuscript ID: PGPH-D-25-03667
Title: Lead poisoning and unique lead sources in Bhutan’s national blood lead level survey, 2024: a nationally representative cross-sectional survey

Dear Dr. Valentina Gallo and Reviewers,

We sincerely thank you for recognizing the merits of our manuscript and for the careful and constructive evaluation provided by you and the three reviewers. We greatly appreciate the detailed and insightful feedback, which has substantially strengthened the methodological transparency, statistical reporting, and clarity of interpretation of our work.

We have carefully addressed each comment and revised the manuscript accordingly. Major revisions include:

- Expanded and clarified description of the complex survey sampling design, including explicit specification of primary sampling units (PSUs), stratification, probability proportional to size (PPS) sampling, and household selection procedures.
- Detailed clarification of the survey-weighted regression framework, variance estimation method, and statistical tests used.
- Improved transparency regarding imputation of values below the limit of detection (LOD), including reporting the proportion imputed and justification of the chosen method.
- Clear distinction between nationally representative and non-representative subgroups.
- Refinement of tables and figures to align with PLOS presentation standards.
- Strengthened discussion of limitations, including cross-sectional design and imputation considerations.
- Clarified Data Availability statement in accordance with PLOS policy.

All changes are reflected in the tracked version of the manuscript.

Below, we respond point-by-point to each reviewer comment.

**Responses to Reviewer #1**

**Comment 1: Title repetition of “lead” and “survey”**

**Response:**
Thank you for this suggestion. The title has been revised to reduce repetition while preserving clarity and specificity. The revised title now reads:

*“Prevalence of lead poisoning and unique lead sources in Bhutan, findings from a nationally represented survey 2024”*

**Comment 2: Clarify arithmetic in abstract (2246 + 1518 > 2959)**

**Response:**
We appreciate this observation. The text has been clarified to indicate that children with BLL ≥5 µg/dL are a subset of those with BLL ≥3.5 µg/dL. The abstract now explicitly states the denominator for both thresholds to avoid confusion.

**Comment 3: Clarify whether *Jinlab Mendrup* analysis was univariate or multivariate; p-value >0.005**

**Response:**
The association between *Jinlab Mendrup* use and elevated BLL was estimated using survey-weighted logistic regression adjusted a priori for child age and sex. This has now been explicitly stated in the Methods section.

Regarding the p-value threshold, we used the conventional α = 0.05 for statistical significance. The value 0.005 is not the accepted universal standard; therefore, we retained the conventional threshold while reporting p-values.

**Comment 4: Provide ethics approval reference**

**Response:**
The Research Ethics Board of Health approval number has now been included in the Methods section.

**Comment 5: Clarify ethical procedures involving children**

**Response:**
We have clarified that written informed consent was obtained from parents or legal guardians for all participating children. Interviews were conducted with caregivers, not children directly. This information has been added to the Methods section.

**Comment 6: Specify statistical tests used**

**Response:**
The Statistical Analysis section has been substantially revised to explicitly state:

- Survey-weighted binary logistic regression was used.
- The complex survey design was accounted for using Taylor series linearized variance estimation.
- P-values were derived from design-adjusted Wald tests.
- For categorical variables with more than two levels, joint Wald tests were used to derive a single global p-value.

**Comment 7: Why not test household water?**

**Response:**
The environmental testing strategy focused on likely high-risk exposure sources identified during field visits and resource feasibility considerations. Water testing was not included due to logistical constraints and national water monitoring data indicating low lead contamination risk. This clarification has been added to the Methods and Discussion sections.

**Comment 8: Tables not suitable for scientific article**

**Response:**
Tables have been reformatted to improve clarity and readability. Redundant columns were removed, formatting standardized, and supplementary tables created where appropriate to reduce density in the main manuscript.

**Comment 9: Avoid figure in Discussion**

**Response:**
The referenced figure has been removed from the Discussion section in accordance with the recommendation.

**Responses to Reviewer #2**

**Comment 1: Clarify representativeness of monastic children and pregnant/breastfeeding women**

**Response:**
We have now explicitly stated in the Results and Discussion sections that findings for monastic children and pregnant/breastfeeding women are not nationally representative and should not be generalized beyond the sampled populations.

**Comment 2: Report proportion below LOD and justify imputation**

**Response:**
We now report that 21.5% of BLL values were below the LeadCare II analyzer’s LOD (3.3 µg/dL). These were treated as left-censored and imputed once using a truncated normal distribution bounded between 0 and 3.299 µg/dL.

We clarified that this approach was chosen over simple substitution (e.g., LOD/2) to better preserve distributional characteristics. Because the LOD was below the primary analytical threshold (≥3.5 µg/dL), classification of children above threshold was not altered by the imputation procedure.

**Comment 3: Acknowledge XRF selection bias**

**Response:**
We have explicitly stated that XRF testing prioritized children with higher BLLs and therefore does not represent population-wide exposure distributions. These results are interpreted as identifying potential exposure sources rather than estimating population prevalence of contaminated items.

**Comment 4: Emphasize association rather than causation (*Jinlab Mendrup*)**

**Response:**
We have revised the Discussion to emphasize that findings demonstrate association rather than causation and expanded discussion of potential confounding and the need for confirmatory laboratory testing.

**Comment 5: Clarify Data Availability statement**

**Response:**
The Data Availability Statement has been revised to clarify that de-identified data may be made available upon reasonable request to the Ministry of Health, subject to ethical approval and data governance procedures.

**Responses to Reviewer #3**

We thank Reviewer #3 for the detailed methodological feedback. The manuscript has been substantially revised to address these concerns.

**Major Comment 1: Complex survey design not adequately described**

**Response:**
We have expanded the Study Design section to explicitly describe:

- Primary Sampling Units (Enumeration Areas)
- Stratification by rural/urban within Dzongkhag and Thromde
- Probability proportional to size (PPS) sampling at the first stage
- Household selection using circular systematic sampling
- Number of households selected and response rates

In the Statistical Analysis section, we now explicitly state that the dataset was survey-set prior to analysis using PSUs, strata, sampling weights, and finite population correction (FPC). All regression analyses used survey-weighted logistic regression with Taylor series linearized variance estimation to account for clustering and stratification.

**Major Comment 2: Ambiguous regression outcome and imputation of outcome**

**Response:**
The regression outcomes were binary indicators of BLL ≥3.5 µg/dL and ≥5 µg/dL, defined in accordance with CDC and WHO reference thresholds. This has been clarified in the Methods.

Values below the LOD were treated as left-censored measurements rather than missing data. A bounded truncated normal imputation was used to preserve distributional properties. Because the LOD (3.3 µg/dL) was below the ≥3.5 µg/dL threshold, imputation did not alter classification of children above threshold.

We acknowledge in the Discussion that imputation introduces uncertainty in estimation of mean BLL.

**Major Comment 3: Presentation of p-values for categorical predictors**

**Response:**
Category-specific inference is conveyed through the 95% confidence intervals for each odds ratio. For variables with more than two categories, a single overall p-value was derived from a joint design-adjusted Wald test assessing the significance of all categories simultaneously. This approach avoids redundancy and ensures the p-value is independent of reference category selection. The Methods section has been revised to clarify this.

**Minor Comment: Regression tables dense**

**Response:**
Tables have been reformatted for improved readability, and selected secondary analyses have been moved to supplementary material.

**Editorial and Administrative Requirements**

- Inclusivity questionnaire completed and uploaded as Supporting Information.
- Financial Disclosure statement revised in full sentences with author initials and funder roles specified.
- Competing Interests statement updated.
- **Fig 2.** Map base layer source is from the [Bhutan National Spatial Data Infrastructure (NSDI)](https://nsdi.systems.gov.bt/data/Boundaries). Data is used under the **'Open' category** with attribution to the [National Land Commission Secretariat (NLCS)](https://web.nlcs.gov.bt/).
- Data Availability statement revised to align with PLOS transparency requirements.

We thank the Editor and Reviewers for their constructive feedback, which has substantially strengthened the methodological clarity and transparency of the manuscript.

Sincerely,

Mongal Singh Gurung
On behalf of all authors
