# Supplementary material for: Prevalence of lead poisoning and unique lead sources in Bhutan: Findings from a nationally representative survey 2024
Source: PLOS Glob Public Health. 2026 Jul 22;6(7):e0006877. doi: 10.1371/journal.pgph.0006877 (PMC13390818; doi:10.1371/journal.pgph.0006877)
Supplement: Attachment — Submitted filename: Response_to_Reviewers_auresp_2.docx [file pgph.0006877.s003.docx]

Manuscript ID: PGPH-D-25-03667

Title: Prevalence of elevated blood lead levels and unique lead sources in Bhutan: findings from a nationally representative survey 2024

Dear Dr. Valentina Gallo and Reviewers,

We sincerely thank you and the reviewers for the careful re-evaluation of our revised manuscript and for the constructive and insightful comments. We are encouraged that the reviewers acknowledged that the concerns raised in the previous round have been adequately addressed.

We appreciate the additional suggestions provided in this round, which have helped us further improve the clarity, transparency, and scientific rigor of the manuscript. In response, we have made targeted revisions to strengthen the reporting of informed consent procedures, refine terminology, enhance methodological transparency (including sampling and statistical analysis), improve the Data Availability Statement, and clarify the interpretation of environmental source testing findings and measurement limitations.

All revisions have been incorporated into the manuscript and are clearly indicated using track changes.

Please find below our point-by-point responses.

**Reviewer #1**

**Comment 1** Please specify how consent was obtained, given that children are involved in the study.

**Response**

We thank the reviewer for this important comment.

We have clarified the informed consent procedures in the Methods section. Specifically, for children aged 1–6 years, written informed consent was obtained from a parent or legal guardian prior to participation, including interview, blood sample collection, and environmental assessment.

For children residing in monastic institutions, consent was obtained from the designated institutional guardian or responsible authority. Assent was obtained from older children where appropriate.

This clarification has been incorporated into the revised manuscript.

**Reviewer #2**

**Comment 1**: Data availability

**Response**

We thank the reviewer for highlighting this important issue.

We have revised the Data Availability Statement to improve clarity and alignment with PLOS data sharing requirements. The dataset contains sensitive individual-level health information collected under the stewardship of the Ministry of Health, Bhutan, and cannot be made publicly available without appropriate safeguards to protect participant confidentiality.

The Ministry of Health is currently developing a comprehensive data governance framework and plans to make de-identified public use datasets available through a national data archival system in the future. At present, the study team is not authorized to publicly release the dataset.

However, the data are available to qualified researchers upon reasonable request for methodologically sound analyses, subject to standard institutional and ethical approval procedures. Requests for data access may be directed to the Ministry of Health, Bhutan at [ppd@health.gov.bt](mailto:ppd@health.gov.bt)

**Comment 2**: Distinction between nationally representative and non-probabilistic samples

**Response**

We thank the reviewer for this important clarification.

We have strengthened the manuscript to clearly distinguish nationally representative findings from those derived from purposive or targeted sampling. Specifically, we now explicitly state that only estimates for children aged 1–6 years are nationally representative.

Findings from monastic children, pregnant or breastfeeding women, and environmental source testing are now clearly described as originating from purposively selected or high-risk populations and should not be interpreted as nationally representative.

This distinction has been reinforced in the Abstract, Results, and Discussion sections.

**Comment 3**: Statistical analysis clarity

**Response**

We thank the reviewer for this important comment.

We have revised the Statistical Analysis section to provide a clearer and more explicit description of the modelling approach and covariate selection. Specifically, we now clarify that all regression models were minimally adjusted for child age and sex, which were selected a priori based on their established role as key demographic confounders associated with both lead exposure and pediatric health outcomes.

We further clarify that fully adjusted multivariable models were not fitted, as the objective of the analysis was to estimate interpretable associations rather than causal effects. Each exposure variable was therefore evaluated in separate models adjusted for age and sex, to avoid overadjustment and to ensure clear interpretation of associations.

In addition, we have clarified that all analyses accounted for the complex survey design, including clustering, stratification, and sampling weights, with variance estimation based on Taylor series linearization.

These revisions improve the transparency, methodological clarity, and interpretability of the analytical approach.

**Comment 4**: LeadCare II and measurement limitations

**Response**

We thank the reviewer for this valuable suggestion.

We have expanded the limitations section to explicitly describe the limitations of the LeadCare II analyzer, including its lower limit of detection and the potential for contamination associated with capillary blood sampling. We also clarify that, although quality control procedures including using D- wipe towels (ESCA Tech) were implemented, these limitations should be considered when interpreting the findings.

Additionally, we note that XRF measurements identify the presence of lead in environmental materials but do not establish causal relationships between specific sources and observed blood lead levels.

We added more details on the implications of public health policy in the discussion. Comparison with studies from other countries is limited as nationally representative data is very scarce.

**Reviewer #3**

**Comment 1**: Title

**Response**

We thank the reviewer for this suggestion.

The title has been revised to improve clarity and align with standard epidemiological terminology:

“Prevalence of lead poisoning and unique lead sources in Bhutan: findings from a nationally representative survey 2024”

**Comment 2**: Use of “lead poisoning” terminology

**Response**

We thank the reviewer for this important comment regarding the use of terminology surrounding lead poisoning. We recognize that the term “lead poisoning” has been used in contradictory ways in literature and that its definition is evolving. While it has been used to refer to toxic levels of lead that require urgent hospitalization and chelation, there is growing use of the term to refer to all blood lead levels since there is no safe level of lead in the body. In that respect, even the term “elevated” is problematic, especially as IQ loss has been shown to occur at levels below public health thresholds.

We acknowledge that the thresholds used in our study (≥3.5 µg/dL and ≥5 µg/dL) are public health reference values, as defined by the Centers for Disease Control and Prevention and WHO, rather than clinical diagnostic thresholds for overt poisoning. We have clarified the use of the two reference thresholds to facilitate comparison with global data. As this study is primarily concerned with the consequences of low-level lead poisoning, we have more precisely defined our uses of the term.

In response, we have revised the manuscript accordingly:

- Instead of using the term “elevated blood lead level,” we have continued to use the term “lead poisoning.”
- We clarified in the abstract that lead causes harm at low levels and that low-level lead poisoning is pervasive.
- The term “lead poisoning” in the Introduction and Discussion sections, is explicitly defined to reflect the continuum of toxicity associated with lead exposure, consistent with evidence from the World Health Organization that no safe blood lead level exists.
- A clarifying statement has been added in the Introduction to explicitly distinguish between epidemiological thresholds used for analysis and the broader toxicological interpretation of lead exposure.

These revisions ensure that the manuscript maintains epidemiological precision while appropriately conveying the public health significance of widespread lead exposure.

We hope this addresses the reviewer’s concern and improves the clarity and interpretability of the manuscript.

**Comment 3**: Sample size transparency

**Response**

We thank the reviewer for this important comment.

We have further strengthened the sample size section by explicitly describing the calculation steps, including assumptions, survey domains, design effect, response rate adjustment, and justification for not applying finite population correction. We have also referenced the publicly available NHS 2023 report for full sampling details.

**Comment 4**: Interpretation of XRF findings

**Response**

We thank the reviewer for this important clarification.

We have revised the Results and Discussion sections to explicitly state that environmental source testing using XRF was conducted in purposively selected high-risk households and institutions. These findings are therefore not nationally representative and should not be interpreted as reflecting population-level prevalence of environmental lead sources.

We now clearly emphasize that these findings are intended to identify potential exposure pathways in high-risk settings.

**Comment 5**: Weighting procedure insufficiently described

**Response**

We thank the reviewer for this important comment. We have strengthened the Statistical Analysis section to provide a clearer description of the weighting procedure. Specifically, we now explicitly state that sampling weights were derived from the NHS 2023 as inverse probabilities of selection across sampling stages, adjusted for household and individual non-response, and normalized for analysis. We have also clarified the application of finite population correction and variance estimation methods. These revisions improve transparency and reproducibility of the national estimates.

**Comment 6**: Typo

**Response**

We thank the reviewer for noting this typographical error. The notation has been corrected to “p < 0.001” in the revised manuscript.

**Conclusion**

We once again thank the Editor and Reviewers for their thoughtful and constructive feedback. We believe that these revisions have substantially improved the clarity, transparency, and scientific rigor of the manuscript.

We hope that the revised manuscript is now suitable for publication in PLOS Global Public Health.

Sincerely,

Mongal Singh Gurung

On behalf of all authors
