# Supplementary material for: Prevalence of lead poisoning and unique lead sources in Bhutan: Findings from a nationally representative survey 2024
Source: PLOS Glob Public Health. 2026 Jul 22;6(7):e0006877. doi: 10.1371/journal.pgph.0006877 (PMC13390818; doi:10.1371/journal.pgph.0006877)
Supplement: Attachment — Submitted filename: Response_to_Reviewers_auresp_3.docx [file pgph.0006877.s004.docx]

Response to Reviewers

Manuscript ID: PGPH-D-25-03667R2

Title: Prevalence of lead poisoning and unique lead sources in Bhutan: findings from a nationally representative survey, 2024

Dear Dr. Desai and Reviewers,

We sincerely thank you and Reviewers for their careful evaluation of our revised manuscript. We are grateful for the positive assessment that the manuscript has substantially improved and that comments from previous review rounds have been adequately addressed.

In response to the remaining comments, we have further strengthened the manuscript by improving the transparency of the blood lead level imputation procedures, clarifying interpretation of regression estimates, refining discussion of potential exposure pathways, strengthening justification for environmental source comparisons, and correcting several presentation and terminology issues. All revisions have been incorporated into the revised manuscript and are highlighted using track changes.

Our point-by-point responses are provided below.

## Reviewer #1

Comment:
The dataset contains sensitive individual-level health information collected under the stewardship of the Ministry of Health, Bhutan, and cannot be made publicly available without appropriate safeguards to protect participant confidentiality. Nonetheless, the Ministry of Health is currently developing a comprehensive data governance framework and plans to make de-identified public use datasets available through a national data archival system in the future. At present, the study team is not authorized to publicly release the dataset. However, the data are available to qualified researchers upon reasonable request for methodologically sound analyses, subject to standard institutional and ethical approval procedures. Requests for data access may be directed to the Ministry of Health, Bhutan at ppd@health.gov.bt

Response:

We thank the reviewer for highlighting this important issue.

We have revised the Data Availability Statement to improve transparency and alignment with PLOS data-sharing requirements. The dataset contains sensitive individual-level health information collected under the stewardship of the Ministry of Health, Bhutan, and cannot be publicly released because of participant confidentiality considerations and national data governance requirements.

The revised statement now clarifies that requests for access to de-identified data may be submitted to the Policy and Planning Division, Ministry of Health, Bhutan ([ppd@health.gov.bt](mailto:ppd@health.gov.bt)). Data may be made available to qualified researchers for methodologically sound analyses, subject to institutional review and applicable ethical approval requirements.

## Reviewer #3

Comment 1:
The imputation procedure for values below the LeadCare II limit of detection requires greater rigor. The authors should provide sensitivity analyses comparing the truncated normal approach with alternatives such as LOD/2 and LOD/√2 and make clear that the mean BLL is model-dependent because of left-censoring.

Response:

We thank the reviewer for this valuable suggestion.

To assess the robustness of the imputation approach, we conducted sensitivity analyses using two commonly applied substitution methods for observations below the limit of detection (LOD): LOD/2 and LOD/√2.

The survey-weighted mean BLL estimates were 5.31 µg/dL (95% CI: 5.17, 5.45) using LOD/2 substitution and 5.46 µg/dL (95% CI: 5.32, 5.59) using LOD/√2 substitution, compared with 5.39 µg/dL (95% CI: 5.25, 5.53) using the primary truncated normal imputation approach. These very similar estimates indicate that the mean BLL was robust to alternative methods for handling observations below the detection limit.

We have added the sensitivity analysis to the Statistical Analysis, Results, and Discussion sections.

Comment 2:
Odds ratios may overstate prevalence ratios when outcomes are common.

Response:

We thank the reviewer for this important methodological observation.

We agree that odds ratios may overestimate prevalence ratios when outcomes are common. Logistic regression was retained because it remains a widely used and well-established method for analysing binary outcomes within complex survey designs and permits straightforward incorporation of sampling weights, clustering, and stratification.

To address the reviewer's concern, we have added clarification in the Statistical Analysis and Discussion sections stating that the reported adjusted odds ratios should be interpreted as measures of association rather than prevalence ratios, or risk ratios. We also explicitly note that the magnitude of odds ratios may overestimate corresponding prevalence ratios because elevated blood lead levels were common in the study population.

Comment 3:
The manuscript should avoid implying that the study has definitively established *Jinlab Mendrup* as a causal source of lead poisoning.

Response:

We thank the reviewer for this important comment.

We agree that the cross-sectional design does not permit causal inference. Accordingly, we revised the manuscript throughout to avoid causal language and to clearly describe *Jinlab Mendrup* as a potential source of lead exposure rather than a confirmed causal determinant of elevated blood lead levels.

We further clarified that although the observed associations, biological plausibility, and detection of lead in *Jinlab Mendrup* samples are consistent with a potential exposure pathway, the study cannot establish causality. Statements implying direct causation have been revised accordingly.

Comment 4:
The manuscript generalizes findings beyond Bhutan without direct supporting evidence.

Response:

We thank the reviewer for this observation.

We have revised the Discussion section to avoid unsupported extrapolation beyond the study setting. Rather than stating that this exposure pathway likely extends beyond Bhutan, we now note that future studies in other Himalayan and Tibetan Buddhist communities may be warranted to determine whether similar contamination exists elsewhere.

Comment 5:
The regression table caption is inaccurate, and there are several presentation and typographical issues, including table numbering inconsistencies and use of the term “convenient sampling”.

Response:

We thank the reviewer for identifying these issues.

We have revised the regression table caption to accurately reflect the analyses presented, corrected typographical errors, resolved table numbering inconsistencies, and replaced the term “convenient sampling” with the standard methodological term “convenience sampling” throughout the manuscript, including the Abstract and Methods sections.

Comment 6:
Interpretation of religious practices should be more evidence-based and cautious.

Response:

We thank the reviewer for this valuable suggestion.

We have revised the relevant discussion to avoid implying a confirmed exposure mechanism. Statements regarding religious items and practices are now presented as plausible hypotheses rather than established pathways. We clarify that the contribution of these items to blood lead levels could not be directly quantified in the present study and warrants further investigation.

At the same time, because lead was detected in several religious and traditional items, we retained recommendations promoting hand hygiene as a precautionary public health measure while additional studies investigate potential exposure pathways.

Comment 7:
The rationale for applying Bangladesh spice thresholds to *Jinlab Mendrup* should be clarified.

Response:

We thank the reviewer for this helpful suggestion.

We have expanded the Methods section to clarify that no specific regulatory threshold currently exists for lead content in *Jinlab Mendrup*. Therefore, the Bangladesh standard for spices was used as the most relevant available comparator for an orally consumed product. We further clarify that threshold exceedance should not be interpreted as evidence of regulatory non-compliance, but rather as a screening benchmark to help identify samples that may warrant further investigation.

Once again, we sincerely thank the Editor and Reviewers for their constructive feedback. We believe that these revisions have strengthened the methodological transparency, scientific rigor, and interpretation of the manuscript and have improved its contribution to the evidence base on childhood lead exposure and environmental health.

Sincerely,

Mongal Singh Gurung
On behalf of all authors
