# Supplementary material for: Prevalence of lead poisoning and unique lead sources in Bhutan: Findings from a nationally representative survey 2024
Source: PLOS Glob Public Health. 2026 Jul 22;6(7):e0006877. doi: 10.1371/journal.pgph.0006877 (PMC13390818; doi:10.1371/journal.pgph.0006877)
Supplement: Peer review history

# Peer review: Decision Letter 0

**DOI:** 10.1371/journal.pgph.0006877.r001

**Authors:** Valentina Gallo

6 Jan 2026

PGPH-D-25-03667

Lead poisoning and unique lead sources in Bhutan’s national blood lead level survey, 2024, a nationally represented cross-sectional survey

PLOS Global Public Health

Dear Dr. Gurung,

Thank you for submitting your manuscript to PLOS Global Public Health. After careful consideration, we feel that it has merit but does not fully meet PLOS Global Public Health’s publication criteria as it currently stands. Therefore, we invite you to submit a revised version of the manuscript that addresses the points raised during the review process.

Particular attention should be paid in clearly and transparently report on study design and statistical analysis. Following the reviewers' advice, please report a detailed description of sampling and statistical methods used. Moreover, please appropriately address strengths and limitations of these choices in the discussion.

Please submit your revised manuscript by Feb 20 2026 11:59PM. If you will need more time than this to complete your revisions, please reply to this message or contact the journal office at globalpubhealth@plos.org. When you're ready to submit your revision, log on to https://www.editorialmanager.com/pgph/ and select the 'Submissions Needing Revision' folder to locate your manuscript file.

Please include the following items when submitting your revised manuscript:

- A letter that responds to each point raised by the editor and reviewer(s). You should upload this letter as a separate file labeled 'Response to Reviewers'.
- A marked-up copy of your manuscript that highlights changes made to the original version. You should upload this as a separate file labeled 'Revised Manuscript with Track Changes'.
- An unmarked version of your revised paper without tracked changes. You should upload this as a separate file labeled 'Manuscript'.

Guidelines for resubmitting your figure files are available below the reviewer comments at the end of this letter.

We look forward to receiving your revised manuscript.

Kind regards,

Valentina Gallo

Academic Editor

PLOS Global Public Health

Journal Requirements:

1. Please include a complete copy of PLOS’ questionnaire on inclusivity in global research in your revised manuscript. Our policy for research in this area aims to improve transparency in the reporting of research performed outside of researchers’ own country or community. The policy applies to researchers who have travelled to a different country to conduct research, research with Indigenous populations or their lands, and research on cultural artefacts. The questionnaire can also be requested at the journal’s discretion for any other submissions, even if these conditions are not met.  Please find more information on the policy and a link to download a blank copy of the questionnaire here: https://journals.plos.org/globalpublichealth/s/best-practices-in-research-reporting. Please upload a completed version of your questionnaire as Supporting Information when you resubmit your manuscript.

2. Please provide a detailed online Financial Disclosure statement. This is published with the article. It must therefore be completed in full sentences and contain the exact wording you wish to be published.

a) State the initials, alongside each funding source, of each author to receive each grant. For example: “This work was supported by the National Institutes of Health (####### to AM; ###### to CJ) and the National Science Foundation (###### to AM).”

b) State what role the funders took in the study. If the funders had no role in your study, please state: “The funders had no role in study design, data collection and analysis, decision to publish, or preparation of the manuscript.”

For more information, please go to our submission guidelines:

https://journals.plos.org/globalpublichealth/s/submission-guidelines#loc-financial-disclosure-statement

3. Please ensure that the funders and grant numbers match between the Financial Disclosure field and the Funding Information tab in your submission form. Note that the funders must be provided in the same order in both places as well.

4. Please update your online Competing Interests statement. If you have no competing interests to declare, please state: “The authors have declared that no competing interests exist.”

5. Some material included in your submission may be copyrighted. According to PLOS’s copyright policy, authors who use figures or other material (e.g., graphics, clipart, maps) from another author or copyright holder must demonstrate or obtain permission to publish this material under the Creative Commons Attribution 4.0 International (CC BY 4.0) License used by PLOS journals. Please closely review the details of PLOS’s copyright requirements here: PLOS Licenses and Copyright. If you need to request permissions from a copyright holder, you may use PLOS's Copyright Content Permission form.

Please respond directly to this email or email the journal office and provide any known details concerning your material's license terms and permissions required for reuse, even if you have not yet obtained copyright permissions or are unsure of your material's copyright compatibility.

Potential Copyright Issues:

Figure 3: Please confirm (a) that you are the photographer; or (b) provide written permission from the photographer to publish the photo(s) under our CC-BY 4.0 license.

Figure 2: please (a) provide a direct link to the base layer of the map (i.e., the country or region border shape) and ensure this is also included in the figure legend; and (b) provide a link to the terms of use / license information for the base layer image or shapefile. We cannot publish proprietary or copyrighted maps (e.g. Google Maps, Mapquest) and the terms of use for your map base layer must be compatible with our CC-BY 4.0 license.

Note: if you created the map in a software program like R or ArcGIS, please locate and indicate the source of the basemap shapefile onto which data has been plotted.

If your map was obtained from a copyrighted source please amend the figure so that the base map used is from an openly available source. Alternatively, please provide explicit written permission from the copyright holder granting you the right to publish the material under our CC-BY 4.0 license.

Please note that the following CC BY licenses are compatible with PLOS license: CC BY 4.0, CC BY 2.0 and CC BY 3.0, meanwhile such licenses as CC BY-ND 3.0 and others are not compatible due to additional restrictions.

If you are unsure whether you can use a map or not, please do reach out and we will be able to help you. The following websites are good examples of where you can source open access or public domain maps:

* U.S. Geological Survey (USGS) - All maps are in the public domain. (http://www.usgs.gov)

* PlaniGlobe - All maps are published under a Creative Commons license so please cite “PlaniGlobe, http://www.planiglobe.com, CC BY 2.0” in the image credit after the caption. (http://www.planiglobe.com/?lang=enl)

* Natural Earth - All maps are public domain. (http://www.naturalearthdata.com/about/terms-of-use/)

6. If the reviewer comments include a recommendation to cite specific previously published works, please review and evaluate these publications to determine whether they are relevant and should be cited. There is no requirement to cite these works unless the editor has indicated otherwise.

Additional Editor Comments (if provided):

[Note: HTML markup is below. Please do not edit.]

Reviewers' comments:

Reviewer's Responses to Questions

**Comments to the Author**

1. Does this manuscript meet PLOS Global Public Health’s publication criteria? Is the manuscript technically sound, and do the data support the conclusions? The manuscript must describe methodologically and ethically rigorous research with conclusions that are appropriately drawn based on the data presented.

Reviewer #1: Yes

Reviewer #2: Yes

Reviewer #3: Partly

**********

2. Has the statistical analysis been performed appropriately and rigorously?

Reviewer #1: Yes

Reviewer #2: Yes

Reviewer #3: No

**********

3. Have the authors made all data underlying the findings in their manuscript fully available (please refer to the Data Availability Statement at the start of the manuscript PDF file)?

The PLOS Data policy requires authors to make all data underlying the findings described in their manuscript fully available without restriction, with rare exception. The data should be provided as part of the manuscript or its supporting information, or deposited to a public repository. For example, in addition to summary statistics, the data points behind means, medians and variance measures should be available. If there are restrictions on publicly sharing data—e.g. participant privacy or use of data from a third party—those must be specified.

Reviewer #1: No

Reviewer #2: Yes

Reviewer #3: No

**********

4. Is the manuscript presented in an intelligible fashion and written in standard English?

PLOS Global Public Health does not copyedit accepted manuscripts, so the language in submitted articles must be clear, correct, and unambiguous. Any typographical or grammatical errors should be corrected at revision, so please note any specific errors here.

Reviewer #1: Yes

Reviewer #2: Yes

Reviewer #3: No

**********

5. Review Comments to the Author

Please use the space provided to explain your answers to the questions above. You may also include additional comments for the author, including concerns about dual publication, research ethics, or publication ethics. (Please upload your review as an attachment if it exceeds 20,000 characters)

Reviewer #1: Title:

there are some repetitions in the title. Two times “lead” and “survey”

I propose to rewrite the title to avoid repetitions

Abstract:

• “Among 1–6-year-old children tested nationally, 2,246/2,959 (75.9% [95% CI 74.0-77.7]) had a BLL≥3.5 μg/dL, and 1,518 (51.3% [95% CI 49.0-53.6]) had a BLL ≥5 μg/dL.”

2246 + 1518 = 3764 > 2959. Can you clarify this information and make it clearer in the summary?

• “Taking Jinlab Mendrup (blessed religious substance) in the previous 15 days was significantly associated with having a BLL ≥3.5 μg/dL, aOR=1.94 (1.34-2.70,p=0.009)”

Is this a univariate or multivariate analysis?

Why is the p-value higher than 0.005, which is the accepted standard?

Introduction

Ok

Methods

• Lines 103-104 “ethical clearance was granted by the Research Ethics Board of Health, Bhutan”

Can you add the reference for the ethics approval that allowed the study to be conducted?

• Line 108” Each selected child’s home was contacted and visited. Enumerators”

How were ethical considerations respected in this context, given that children are involved?

Please specify.

Who answered the questions, the child or the parents?

Please specify.

• Lines158-159: “Associations were adjusted for age and sex and presented as adjusted odds ratios (aORs) with 95% CIs and p-values.”

please specify the different statistical tests used, until the associated variables are identified, specifying the chosen p-value each time.

• Why not also test the water available in the homes surveyed?

Results

• Review the presentation of all tables in the document. They are not suitable for a scientific article. This refers to articles already published in PLOS.

Also, in Table 1, the third column is not necessary.

Discussion

• line 317 : It is not normally advisable to include figures in the discussion.

Images

The image quality must be improved.

Reviewer #2: Summary

This manuscript reports findings from Bhutan’s first nationally representative blood lead level (BLL) survey, conducted in 2024, with the additional investigation of potential environmental lead sources. The study addresses a major global public health concern and provides rare nationally representative data from a low- and middle-income country. The identification of culturally specific lead exposure pathways, particularly Jinlab Mendrup, represents a novel and highly important contribution to the literature. Overall, the manuscript is well written, methodologically sound, and highly relevant to the scope of PLOS Global Public Health. With minor to moderate revisions, it would be suitable for publication.

Major Comments

1. Clarity on Representativeness and Generalizability

While the nationally representative sampling of children aged 1–6 years is clearly described, other study populations (monastic children and pregnant/breastfeeding women) were selected through purposive or convenience sampling. Although this is mentioned in the Methods, the manuscript would benefit from a more explicit and repeated clarification- particularly in the Results and Discussion—that findings from these groups are not nationally representative and should not be generalized beyond the sampled populations.

Recommendation:

Please clearly distinguish nationally representative estimates from non-representative findings in the Results section and reiterate this distinction in the Discussion when interpreting prevalence differences.

2. Blood Lead Measurement and Imputation

The use of capillary blood sampling analyzed with the LeadCare II analyzer is well justified given local constraints. However, the imputation of BLLs below the limit of detection (LOD) using a truncated normal distribution warrants additional transparency.

Recommendations:

• Report the proportion of BLL values below the LOD that required imputation.

• Briefly justify the choice of truncated normal distribution over alternative approaches and discuss its potential impact on prevalence and mean estimates.

3. Environmental Source Testing Strategy

The XRF-based environmental testing provides valuable insight into potential lead sources. However, the selection strategy for households and institutions prioritized children with higher BLLs, which may bias the observed prevalence of lead-containing items.

Recommendation:

Please explicitly acknowledge that XRF findings may overestimate the prevalence of lead-containing items at the population level and clarify that these results identify potential sources rather than population-wide exposure distributions.

4. Interpretation of Jinlab Mendrup Association

The association between Jinlab Mendrup consumption and elevated BLLs is a key and novel finding. While the manuscript appropriately avoids definitive causal claims, some language may still be interpreted as causal.

Recommendations:

• Further, emphasize that the findings demonstrate a strong association rather than causation.

• Expand discussion on potential confounding factors (e.g., socio-cultural practices associated with Jinlab Mendrup use) and the need for confirmatory laboratory analyses of samples.

5. Data Availability Statement

The Data Availability Statement explains that data sharing is restricted due to Ministry of Health ownership. While such restrictions may be acceptable under PLOS policy, the statement should more clearly align with the journal’s transparency requirements.

Recommendation:

Please clarify the conditions under which data access may be granted, emphasizing that access is possible for researchers with methodologically sound proposals and appropriate approvals, rather than implying categorical restriction.

Minor Comments

1. Abstract

• Consider explicitly stating “nationally representative cross-sectional survey” at the beginning of the Methods description for clarity.

• Minor streamlining of technical detail (e.g., XRF thresholds) could improve readability.

2. Terminology and Consistency

• Ensure consistent use of units (µg/dL) and statistical notation throughout the manuscript.

3. Tables and Figures

• Table 3 is extensive; consider moving selected variables or secondary analyses to supplementary material.

• Ensure all figures meet PLOS resolution requirements and that legends are fully self-contained.

4. Discussion

The Discussion is strong; however, a brief policy-oriented paragraph outlining feasible regulatory or public health actions would further align the manuscript with the journal’s global health focus.

Overall Recommendation

Minor to Moderate Revision

This manuscript makes an important and original contribution to global environmental health literature. The study is methodologically rigorous, ethically sound, and highly relevant to public health policy in low-resource settings. Addressing the comments above will further strengthen clarity, transparency, and interpretability, and will enhance the manuscript’s suitability for publication in PLOS Global Public Health.

Reviewer #3: Overall assessment:

This manuscript addresses an important public health problem and tries to present rare nationally representative blood lead level (BLL) data from a low and middle-income country. The scale of data collection and the integration of environmental source testing are notable strengths. However, substantial methodological, statistical, and reporting weaknesses limit interpretability, internal validity, and reproducibility, particularly given the complex survey design and the analytic claims made. Several issues, especially regarding sampling, clustering, regression modeling, outcome definition, weighting, and results presentation, require major revision before the manuscript can be considered for publication.

MAJOR COMMENTS:

1. Inadequate handling and reporting of the complex survey design and clustering in the Materials and Methods section (Study design, Statistical analysis).

The study is described as a “nationally represented cross-sectional subset of the National Health Survey (NHS) 2023” using a multi-stage cluster sampling design (lines 85–86). However, the manuscript does not specify the primary sampling unit (PSU) (e.g., enumeration area, village, household). The number of clusters is not clearly stated, the stratification variables used at analysis is unclear. Whether probability proportional to size (PPS) sampling was used at any stage especially during sampling is not specified, which raises questions if the so called “nationally represented” study is a good reflection of the population the sample was taken. These omissions are critical for a national survey and prevent readers from evaluating design effects and variance estimation.

While the authors state that “all estimates for children aged 1–6 years were weighted using normalized sampling and non-response weights” (Statistical analysis section lines 154-155), it is not stated whether it was also applied in the regression analyses, or if they survey set the data before analysis, accounted for intra-cluster correlation, or Used robust/clustered standard errors at the PSU level, just leaves the reader guessing to fill in the blanks. Further, the regression model fitted is not well explained. This ambiguity forces readers to infer the modeling approach from the presentation of odds ratios, which is unacceptable in a methods section. If its binary logistic regression, state it, if you used the generalized linear modelling approach state it, authors should consider being as transparent as possible in this section for the sake of reproducibility. Clearly describe the full regression model(s) fitted, the covariate selection strategy, whether models were hierarchical, domain-based, or fully adjusted. Explicitly state the statistical test used for each p-value.

Presenting odds ratios with narrow confidence intervals (consistent from abstract to results section) in a multi-stage national survey strongly suggests that clustering may not have been fully accounted for, potentially inflating precision and Type I error. Explicitly describe the survey design structure (PSUs, strata, sampling stages). State clearly whether survey-weighted logistic regression with appropriate variance estimation was used. If clustering was ignored in regression modeling, the analyses must be re-run using survey-adjusted methods, if it was done then clearly explain what you did.

2. Ambiguous and poorly defined regression outcome

The outcome used in regression analyses is described only as “BLL ≥3.5 µg/dL” or “BLL ≥5 µg/dL” (lines 155-157), but the manuscript fails to clearly define what the binary outcome represents conceptually. Author said “BLLs below the LeadCare II analyzer’s level of detection (LOD <3.3 μg/dL) were imputed using a truncated normal distribution.” Lines 153-154, and BLL seems to be the outcome. Generally, it is not good practice to impute values for the outcome variable. Authors must consider a better approach dealing with values for the outcome.

3. Inappropriate and misleading presentation of p-values for categorical predictors (Table 3, throughout)

For nearly all categorical variables (e.g., age, income quintile, housing materials), the Table 3 presents multiple category-specific odds ratios, but a single p-value covering the entire variable. This approach masks heterogeneity of effects across categories, prevents readers from assessing which contrasts are statistically meaningful, it is inconsistent with transparent reporting standards. Revise this

MINOR BUT IMPORTANT REVISIONS:

• Imputation of values below the limit of detection is insufficiently justified. It is unclear whether imputation was performed once or multiple times. Provide a clear rationale for the imputation method.

• Regression tables are excessively dense and difficult to interpret

**********

6. PLOS authors have the option to publish the peer review history of their article (what does this mean?). If published, this will include your full peer review and any attached files.

**Do you want your identity to be public for this peer review?**  If you choose “no”, your identity will remain anonymous but your review may still be made public.

For information about this choice, including consent withdrawal, please see our Privacy Policy.

Reviewer #1: No

Reviewer #2: **Yes:** NICOLE FREITAS

Reviewer #3: No

**********

[NOTE: If reviewer comments were submitted as an attachment file, they will be attached to this email and accessible via the submission site. Please log into your account, locate the manuscript record, and check for the action link "View Attachments". If this link does not appear, there are no attachment files.]

-->Figure Resubmissions:

While revising your submission, we strongly recommend that you use PLOS’s NAAS tool (https://ngplosjournals.pagemajik.ai/artanalysis) to test your figure files. NAAS can convert your figure files to the TIFF file type and meet basic requirements (such as print size, resolution), or provide you with a report on issues that do not meet our requirements and that NAAS cannot fix.

After uploading your figures to PLOS’s NAAS tool - https://ngplosjournals.pagemajik.ai/artanalysis, NAAS will process the files provided and display the results in the "Uploaded Files" section of the page as the processing is complete. If the uploaded figures meet our requirements (or NAAS is able to fix the files to meet our requirements), the figure will be marked as "fixed" above. If NAAS is unable to fix the files, a red "failed" label will appear above. When NAAS has confirmed that the figure files meet our requirements, please download the file via the download option, and include these NAAS processed figure files when submitting your revised manuscript.

# Author comment: Author response to Decision Letter 1

**DOI:** 10.1371/journal.pgph.0006877.r002

2 Mar 2026

Attachment Submitted filename: Response to Reviewers.docx

# Peer review: Decision Letter 1

**DOI:** 10.1371/journal.pgph.0006877.r003

**Authors:** Valentina Gallo

26 Mar 2026

PGPH-D-25-03667R1

Prevalence of lead poisoning and unique lead sources in Bhutan, findings from a nationally represented survey 2024

PLOS Global Public Health

Dear Dr. Gurung,

Thank you for submitting your manuscript to PLOS Global Public Health. After careful consideration, we feel that it has merit but does not fully meet PLOS Global Public Health’s publication criteria as it currently stands. Therefore, we invite you to submit a revised version of the manuscript that addresses the points raised during the review process.

It is important that you improve the clarity of the reporting of your research in order to maximize transparency. Please follow the helpful suggestions of the reviewers.

Please submit your revised manuscript by Apr 25 2026 11:59PM. If you will need more time than this to complete your revisions, please reply to this message or contact the journal office at globalpubhealth@plos.org. When you're ready to submit your revision, log on to https://www.editorialmanager.com/pgph/ and select the 'Submissions Needing Revision' folder to locate your manuscript file.

Please include the following items when submitting your revised manuscript:

- A letter that responds to each point raised by the editor and reviewer(s). You should upload this letter as a separate file labeled 'Response to Reviewers'.
- A marked-up copy of your manuscript that highlights changes made to the original version. You should upload this as a separate file labeled 'Revised Manuscript with Track Changes'.
- An unmarked version of your revised paper without tracked changes. You should upload this as a separate file labeled 'Manuscript'.

Guidelines for resubmitting your figure files are available below the reviewer comments at the end of this letter.

We look forward to receiving your revised manuscript.

Kind regards,

Valentina Gallo

Academic Editor

PLOS Global Public Health

Journal Requirements:

1. If the reviewer comments include a recommendation to cite specific previously published works, please review and evaluate these publications to determine whether they are relevant and should be cited. There is no requirement to cite these works unless the editor has indicated otherwise.

2. Please review your reference list to ensure that it is complete and correct. If you have cited papers that have been retracted, please include the rationale for doing so in the manuscript text, or remove these references and replace them with relevant current references. Any changes to the reference list should be mentioned in the rebuttal letter that accompanies your revised manuscript. If you need to cite a retracted article, indicate the article’s retracted status in the References list and also include a citation and full reference for the retraction notice.

Additional Editor Comments (if provided):

[Note: HTML markup is below. Please do not edit.]

Reviewers' comments:

Reviewer's Responses to Questions

**Comments to the Author**

1. If the authors have adequately addressed your comments raised in a previous round of review and you feel that this manuscript is now acceptable for publication, you may indicate that here to bypass the “Comments to the Author” section, enter your conflict of interest statement in the “Confidential to Editor” section, and submit your "Accept" recommendation.

Reviewer #1: All comments have been addressed

Reviewer #2: All comments have been addressed

Reviewer #3: All comments have been addressed

**********

2. Does this manuscript meet PLOS Global Public Health’s publication criteria? Is the manuscript technically sound, and do the data support the conclusions? The manuscript must describe methodologically and ethically rigorous research with conclusions that are appropriately drawn based on the data presented.

Reviewer #1: Yes

Reviewer #2: Partly

Reviewer #3: Partly

**********

3. Has the statistical analysis been performed appropriately and rigorously?

Reviewer #1: Yes

Reviewer #2: Yes

Reviewer #3: Yes

**********

4. Have the authors made all data underlying the findings in their manuscript fully available (please refer to the Data Availability Statement at the start of the manuscript PDF file)?

The PLOS Data policy requires authors to make all data underlying the findings described in their manuscript fully available without restriction, with rare exception. The data should be provided as part of the manuscript or its supporting information, or deposited to a public repository. For example, in addition to summary statistics, the data points behind means, medians and variance measures should be available. If there are restrictions on publicly sharing data—e.g. participant privacy or use of data from a third party—those must be specified.

Reviewer #1: Yes

Reviewer #2: No

Reviewer #3: No

**********

5. Is the manuscript presented in an intelligible fashion and written in standard English?

PLOS Global Public Health does not copyedit accepted manuscripts, so the language in submitted articles must be clear, correct, and unambiguous. Any typographical or grammatical errors should be corrected at revision, so please note any specific errors here.

Reviewer #1: Yes

Reviewer #2: Yes

Reviewer #3: Yes

**********

6. Review Comments to the Author

Please use the space provided to explain your answers to the questions above. You may also include additional comments for the author, including concerns about dual publication, research ethics, or publication ethics. (Please upload your review as an attachment if it exceeds 20,000 characters)

Reviewer #1: Please

specify how consent was obtained, given that children are involved in the study. If consent was obtained from the parents or legal guardians, please specify this. Thank you

Reviewer #2: After carefully reviewing the manuscript, I consider that the study addresses an important global public health issue, namely lead exposure among children. The manuscript presents data from a national survey and integrates both biological and environmental measurements, which represents a significant strength and aligns well with the type of empirical research published in PLOS ONE.

One of the main strengths of the manuscript is the relevance of the topic and the originality of the data. National estimates of blood lead levels in children are rarely available in low- and middle-income countries, and the relatively large sample size strengthens the contribution of this work. In addition, the use of X-ray fluorescence (XRF) to investigate possible environmental sources of lead exposure represents an interesting and relevant methodological approach. The manuscript also reports ethical considerations and data collection procedures with reasonable clarity.

However, several issues should be addressed to improve the transparency and scientific rigor of the study in accordance with PLOS ONE guidelines. First, the data availability statement requires clarification. The manuscript indicates that the dataset cannot be shared directly because it belongs to the Ministry of Health of Bhutan and can only be accessed with institutional approval. The authors should clarify these restrictions more clearly and, if possible, consider depositing the dataset in a public repository or explicitly describing the procedure for requesting access.

Second, the representativeness of the sample should be more clearly explained. The study includes three population groups (young children, children in monastic institutions, and pregnant or breastfeeding women), but only the sample of children aged 1–6 years appears to be nationally representative. The authors should clearly distinguish between nationally representative findings and results derived from non-probabilistic samples.

Third, the description of the statistical analysis could be improved. The manuscript reports associations between several factors and elevated blood lead levels, but it is not entirely clear which covariates were included in the adjusted models or how potential confounders were selected. Providing a clearer description of the analytical approach would strengthen the interpretation of the findings.

In addition, the limitations of the measurement methods should be discussed in greater detail. Blood lead levels were measured using the LeadCare II system, which is commonly used in field studies but has known limitations, including detection thresholds and potential contamination when capillary samples are used. These limitations should be acknowledged and discussed more explicitly. Similarly, environmental measurements obtained through XRF should be interpreted cautiously, as the presence of lead in a material does not necessarily imply a direct causal relationship with the blood lead levels observed in children.

The manuscript generally follows an appropriate scientific structure, although some sections could be streamlined for clarity. The abstract could briefly specify the study design and clarify which findings are nationally representative. The discussion would also benefit from stronger comparison with studies from other countries and a clearer articulation of the implications for public health policy.

Overall, the manuscript addresses an important topic and provides valuable data on lead exposure in children. However, improvements in methodological clarity, data availability transparency, and discussion of study limitations are necessary to strengthen the scientific quality of the work.

Reviewer #3: This manuscript presents findings from a nationally representative blood lead level (BLL) survey, this is an important and under-studied public health issue. Good job working on the initial comments, just a few final touches remain, several sections require clarification, methodological justification, and improved reporting to meet the standards of transparency and reproducibility expected in scientific publications. Here are some of the considerations:

Lines 2–4, title of article is “Prevalence of lead poisoning and unique lead sources in Bhutan, findings from a nationally represented survey 2024” The phrase “nationally represented survey” sounds grammatically incorrect and uncommon in epidemiologic literature. The common terminology is “nationally representative survey.” Authors can consider rephrasing the title in that regard.

Between lines 38–44, the terminology “lead poisoning” vs “elevated blood lead levels”, authors said “Lead poisoning prevalence was high across all ages, rural/urban locations, and income quintiles.” However, the study defines thresholds of ≥3.5 µg/dL and ≥5 µg/dL, but these seem to be reference levels rather than clinical poisoning thresholds. Using the phrase “lead poisoning prevalence” may overstate the clinical interpretation. Authors should be carefull with the use of phrases such as “Lead poisoning prevalence was high…” in the entire article without clear justification, consider justifiable statements like “The prevalence of elevated blood lead levels was high…”

Lines 103–108, Authors said “The sample size for the NBLLS 2024 was calculated in three steps… The final target was 3,688.” The calculation is not sufficiently transparent. Key elements are missing such as: design effect, clustering adjustments, whether finite population corrections were applied. Because this is a clustered national survey, a design effect should typically be incorporated.

Consider providing a clearer formula and explanation including.

Lines 159–165, author saidd “XRF testing was offered for the home of every child with a BLL ≥20 µg/dL… the home of the child with the highest BLL ≥10 µg/dL was selected.” This is a targeted sampling strategy, meaning that environmental samples are not representative of national exposure sources. Although the authors briefly acknowledge this (line 165), the Abstract and Discussion appear to imply broader generalizability.Explicitly state in results and discussion that source testing reflects high-risk environments

Lines 188–191; said “The survey design was specified in Stata using primary sampling units (PSUs), stratification… normalized sampling and non-response weights…” The weighting procedure is insufficiently described. Missing details such as how weights were derived from NHS 2023, adjustment for non-response, post-stratification procedures. Provide more details.This is important because the national prevalence estimate is an important finding that other studies may cite.

Line 278, minor typo “p=<0.001”. Consider revising the notation to p < 0.001

**********

7. PLOS authors have the option to publish the peer review history of their article (what does this mean?). If published, this will include your full peer review and any attached files.

**Do you want your identity to be public for this peer review?**  If you choose “no”, your identity will remain anonymous but your review may still be made public.

For information about this choice, including consent withdrawal, please see our Privacy Policy.

Reviewer #1: No

Reviewer #2: No

Reviewer #3: No

**********

[NOTE: If reviewer comments were submitted as an attachment file, they will be attached to this email and accessible via the submission site. Please log into your account, locate the manuscript record, and check for the action link "View Attachments". If this link does not appear, there are no attachment files.]

Figure Resubmissions:

While revising your submission, we strongly recommend that you use PLOS’s NAAS tool (https://ngplosjournals.pagemajik.ai/artanalysis) to test your figure files. NAAS can convert your figure files to the TIFF file type and meet basic requirements (such as print size, resolution), or provide you with a report on issues that do not meet our requirements and that NAAS cannot fix.

After uploading your figures to PLOS’s NAAS tool - https://ngplosjournals.pagemajik.ai/artanalysis, NAAS will process the files provided and display the results in the "Uploaded Files" section of the page as the processing is complete. If the uploaded figures meet our requirements (or NAAS is able to fix the files to meet our requirements), the figure will be marked as "fixed" above. If NAAS is unable to fix the files, a red "failed" label will appear above. When NAAS has confirmed that the figure files meet our requirements, please download the file via the download option, and include these NAAS processed figure files when submitting your revised manuscript.

# Author comment: Author response to Decision Letter 2

**DOI:** 10.1371/journal.pgph.0006877.r004

24 Apr 2026

Attachment Submitted filename: Response_to_Reviewers_auresp_2.docx

# Peer review: Decision Letter 2

**DOI:** 10.1371/journal.pgph.0006877.r005

**Authors:** Gauri Desai

8 Jun 2026

PGPH-D-25-03667R2

Prevalence of lead poisoning and unique lead sources in Bhutan: findings from a nationally representative survey 2024

PLOS Global Public Health

Dear Dr. Gurung,

Thank you for submitting your manuscript to PLOS Global Public Health. After careful consideration, we feel that it has merit but does not fully meet PLOS Global Public Health’s publication criteria as it currently stands. Therefore, we invite you to submit a revised version of the manuscript that addresses the points raised during the review process.

Please submit your revised manuscript by Jul 08 2026 11:59PM. If you will need more time than this to complete your revisions, please reply to this message or contact the journal office at globalpubhealth@plos.org. When you're ready to submit your revision, log on to https://www.editorialmanager.com/pgph/ and select the 'Submissions Needing Revision' folder to locate your manuscript file.

Please include the following items when submitting your revised manuscript:

- A letter that responds to each point raised by the editor and reviewer(s). You should upload this letter as a separate file labeled 'Response to Reviewers'.
- A marked-up copy of your manuscript that highlights changes made to the original version. You should upload this as a separate file labeled 'Revised Manuscript with Track Changes'.
- An unmarked version of your revised paper without tracked changes. You should upload this as a separate file labeled 'Manuscript'.

Guidelines for resubmitting your figure files are available below the reviewer comments at the end of this letter.

As the corresponding author, your ORCID iD is verified in the submission system and will appear in the published article. PLOS supports the use of ORCID, and we encourage all coauthors to register for an ORCID iD and use it as well. Please encourage your coauthors to verify their ORCID iD within the submission system before final acceptance, as unverified ORCID iDs will not appear in the published article. *Only*  the individual author can complete the verification step; PLOS staff *cannot*  verify ORCID iDs on behalf of authors.

We look forward to receiving your revised manuscript.

Kind regards,

Gauri Desai, PhD

Academic Editor

PLOS Global Public Health

Journal Requirements:

If the reviewer comments include a recommendation to cite specific previously published works, please review and evaluate these publications to determine whether they are relevant and should be cited. There is no requirement to cite these works unless the editor has indicated otherwise.

Please review your reference list to ensure that it is complete and correct. If you have cited papers that have been retracted, please include the rationale for doing so in the manuscript text, or remove these references and replace them with relevant current references. Any changes to the reference list should be mentioned in the rebuttal letter that accompanies your revised manuscript. If you need to cite a retracted article, indicate the article’s retracted status in the References list and also include a citation and full reference for the retraction notice.

Additional Editor Comments (if provided):

[Note: HTML markup is below. Please do not edit.]

Reviewers' comments:

Reviewer's Responses to Questions

**Comments to the Author**

1. If the authors have adequately addressed your comments raised in a previous round of review and you feel that this manuscript is now acceptable for publication, you may indicate that here to bypass the “Comments to the Author” section, enter your conflict of interest statement in the “Confidential to Editor” section, and submit your "Accept" recommendation.

Reviewer #1: All comments have been addressed

Reviewer #2: All comments have been addressed

Reviewer #3: All comments have been addressed

**********

2. Does this manuscript meet PLOS Global Public Health’s publication criteria? Is the manuscript technically sound, and do the data support the conclusions? The manuscript must describe methodologically and ethically rigorous research with conclusions that are appropriately drawn based on the data presented.

Reviewer #1: Yes

Reviewer #2: Yes

Reviewer #3: Partly

**********

3. Has the statistical analysis been performed appropriately and rigorously?

Reviewer #1: Yes

Reviewer #2: Yes

Reviewer #3: Yes

**********

4. Have the authors made all data underlying the findings in their manuscript fully available (please refer to the Data Availability Statement at the start of the manuscript PDF file)?

The PLOS Data policy requires authors to make all data underlying the findings described in their manuscript fully available without restriction, with rare exception. The data should be provided as part of the manuscript or its supporting information, or deposited to a public repository. For example, in addition to summary statistics, the data points behind means, medians and variance measures should be available. If there are restrictions on publicly sharing data—e.g. participant privacy or use of data from a third party—those must be specified.

Reviewer #1: No

Reviewer #2: Yes

Reviewer #3: No

**********

5. Is the manuscript presented in an intelligible fashion and written in standard English?

PLOS Global Public Health does not copyedit accepted manuscripts, so the language in submitted articles must be clear, correct, and unambiguous. Any typographical or grammatical errors should be corrected at revision, so please note any specific errors here.

Reviewer #1: Yes

Reviewer #2: Yes

Reviewer #3: Yes

**********

6. Review Comments to the Author

Please use the space provided to explain your answers to the questions above. You may also include additional comments for the author, including concerns about dual publication, research ethics, or publication ethics. (Please upload your review as an attachment if it exceeds 20,000 characters)

Reviewer #1: About the data the author says that "The dataset contains sensitive individual-level health information collected

under the stewardship of the Ministry of Health, Bhutan, and cannot be made publicly available

without appropriate safeguards to protect participant confidentiality"

Reviewer #2: no recommendations for the authors, the comments are well addressed on the manuscript.

Reviewer #3: The manuscript addresses an important and under-studied public health issue, and the nationally representative survey of blood lead levels among young children in Bhutan is potentially a valuable contribution. However, although the manuscript is generally intelligible and the main prevalence finding is important, I do not think all aspects of the manuscript are yet sufficiently rigorous, transparent, or publication ready:

Lines 208–217: The authors state that 21.5% of child BLL measurements were below the Lead Care II limit of detection and that these values were imputed once using a truncated normal distribution. The authors used a single stochastic imputation method for values below the LOD and state that this approach was chosen to preserve distributional properties and reduce bias in estimating mean BLL. This is not sufficiently rigorous as currently presented. A single imputation does not propagate imputation uncertainty into the variance estimates. Since more than one-fifth of child BLL measurements were below the LOD, the reported mean BLL depends materially on this assumption. The authors correctly note that prevalence classification at ≥3.5 µg/dL is not altered by this imputation, but the mean and SD are still affected. The authors should either use multiple imputation or censored-data methods, or at minimum provide sensitivity analyses comparing the truncated normal approach with common alternatives such as LOD/2 and LOD/√2. They should also make clear in the results and discussion that the mean BLL is model-dependent because of left-censoring.

Lines 233–246 and Table 4: The authors use logistic regression for outcomes with very high prevalence. The authors estimate associations with BLL ≥3.5 µg/dL and ≥5 µg/dL using survey-weighted logistic regression and report adjusted odds ratios. Odds ratios are difficult to interpret when outcomes are common. In this study, 75.9% of children had BLL ≥3.5 µg/dL and 51.3% had BLL ≥5 µg/dL. Under these conditions, odds ratios can substantially overstate prevalence ratios or risk ratios. This may make the magnitude of association appear stronger than it is. The authors should either report survey-weighted prevalence ratios, for example using a modified Poisson or log-binomial approach where feasible, or explicitly warn readers that the odds ratios should not be interpreted as risk ratios. This is particularly important for the Jinlab Mendrup finding, which is likely to attract policy and media attention.

Lines 40–43, 324–330, and 447–455: The authors report that recent Jinlab Mendrup use was associated with elevated BLL and that 44.2% of tested Jinlab Mendrup samples contained lead. The authors identify Jinlab Mendrup as a potential risk and state that any lead present in Jinlab Mendrup would contribute directly to lead poisoning because it is consumed. The association is plausible and important, but the language is too strong in places. The cross-sectional design cannot establish temporality or causality. The manuscript does not quantify dose, amount consumed, frequency, bioavailability, or whether the child’s own consumed sample was the contaminated sample tested. In addition, the environmental testing was not population-representative. The authors should should avoid implying that the study has definitively established Jinlab Mendrup as a causal source of lead poisoning. A stronger causal claim would require product-specific exposure assessment, dose estimation, repeated BLL testing, etc.

Lines 447–455: The authors state that because Jinlab Mendrup is used across Himalayan and Tibetan-Buddhist communities globally, this exposure pathway likely extends beyond Bhutan and merits coordinated regional and diaspora-focused mitigation. The authors generalize the potential exposure pathway beyond Bhutan. This statement may be plausible but is not directly supported by the data presented in this manuscript. The study was conducted in Bhutan and does not test Jinlab Mendrup products from India, Nepal, Tibet, Mongolia, or other commutnities. The language risks overgeneralization, especially on a culturally sensitive religious practice.

Lines 332–334: The regression table caption states “crude Odds Ratios and adjusted Odds Ratio,” but the table appears to present adjusted odds ratios only. The table caption suggests that both crude and adjusted odds ratios are reported. This is inaccurate or at least unclear. If crude odds ratios are not shown, the caption is misleading. There is also inconsistent capitalization and singular/plural grammar.

Lines 288–292 and Table 2: The table title says “nationally represented” and the table includes at least one apparent typographical error, “666/ (47.1%).” Table 2 presents prevalence, confidence intervals, and mean BLL by demographics. The table has presentation errors that should not remain in a publication ready manuscript.

Lines 342–352 and later XRF table references: The manuscript appears to have a table numbering inconsistency. The text appears to refer to the XRF results as Table 5, while the displayed heading identifies an environmental source table as Table 4, after the regression table has already been labelled Table 4.

Lines 144–146: The authors use the term “convenient sampling.”

“Convenient sampling” was used for pregnant/breastfeeding women, and monastic institutions were purposively selected. The standard methodological term is “convenience sampling,” not “convenient sampling.” This error also appears in the abstract. Consider replacing “convenient sampling” with “convenience sampling” throughout the manuscript.

Lines 470–475: The authors state that religious and Bhutanese items may transfer lead to hands and that daily religious practices may contribute to exposure. Touching items containing lead could transfer lead to hands, and washing before but not after entering altar rooms may contribute to exposure. This is a plausible hypothesis, but it is speculative. The study did not measure hand lead loading, transfer efficiency, frequency of contact, or post-contact ingestion. Because this involves religious practices, the wording should be especially careful and evidence-bound.

Lines 253–259: The authors state that reference thresholds for XRF data were chosen from international and regional standards and that Bangladesh’s threshold for spices was used for Jinlab Mendrup and spices. Bangladesh’s threshold for spices was applied to both spices and Jinlab Mendrup. Applying a spice threshold to Jinlab Mendrup may be pragmatic, but the rationale is not sufficiently justified. Jinlab Mendrup is a religious substance, not a spice, and the frequency, quantity, and mode of ingestion may differ. The authors should explain why the spice threshold is the most appropriate available comparator, acknowledge the absence of a specific regulatory threshold for Jinlab Mendrup, and avoid presenting threshold exceedance as definitive evidence of regulatory non-compliance unless the threshold legally applies.

**********

7. PLOS authors have the option to publish the peer review history of their article (what does this mean?). If published, this will include your full peer review and any attached files.

**Do you want your identity to be public for this peer review?**  If you choose “no”, your identity will remain anonymous but your review may still be made public.

For information about this choice, including consent withdrawal, please see our Privacy Policy.

Reviewer #1: **Yes:** Karine Lucrèce Marie CODJO-SEIGNON

Reviewer #2: **Yes:** Nicole Freitas

Reviewer #3: No

**********

[NOTE: If reviewer comments were submitted as an attachment file, they will be attached to this email and accessible via the submission site. Please log into your account, locate the manuscript record, and check for the action link "View Attachments". If this link does not appear, there are no attachment files.]

Figure Resubmissions:

While revising your submission, we strongly recommend that you use PLOS’s NAAS tool (https://ngplosjournals.pagemajik.ai/artanalysis) to test your figure files. NAAS can convert your figure files to the TIFF file type and meet basic requirements (such as print size, resolution), or provide you with a report on issues that do not meet our requirements and that NAAS cannot fix.

After uploading your figures to PLOS’s NAAS tool - https://ngplosjournals.pagemajik.ai/artanalysis, NAAS will process the files provided and display the results in the "Uploaded Files" section of the page as the processing is complete. If the uploaded figures meet our requirements (or NAAS is able to fix the files to meet our requirements), the figure will be marked as "fixed" above. If NAAS is unable to fix the files, a red "failed" label will appear above. When NAAS has confirmed that the figure files meet our requirements, please download the file via the download option, and include these NAAS processed figure files when submitting your revised manuscript.

# Author comment: Author response to Decision Letter 3

**DOI:** 10.1371/journal.pgph.0006877.r006

23 Jun 2026

Attachment Submitted filename: Response_to_Reviewers_auresp_3.docx

# Editor report: Decision Letter 3

**DOI:** 10.1371/journal.pgph.0006877.r007

**Authors:** Gauri Desai

29 Jun 2026

Prevalence of lead poisoning and unique lead sources in Bhutan: findings from a nationally representative survey 2024

PGPH-D-25-03667R3

Dear Mr. Gurung,

We are pleased to inform you that your manuscript 'Prevalence of lead poisoning and unique lead sources in Bhutan: findings from a nationally representative survey 2024' has been provisionally accepted for publication in PLOS Global Public Health.

Before your manuscript can be formally accepted you will need to complete some formatting changes, which you will receive in a follow up email. A member of our team will be in touch with a set of requests.

Please note that your manuscript will not be scheduled for publication until you have made the required changes, so a swift response is appreciated.

IMPORTANT: The editorial review process is now complete. PLOS will only permit corrections to spelling, formatting or significant scientific errors from this point onwards. Requests for major changes, or any which affect the scientific understanding of your work, will cause delays to the publication date of your manuscript.

If your institution or institutions have a press office, please notify them about your upcoming paper to help maximize its impact. If they'll be preparing press materials, please inform our press team as soon as possible -- no later than 48 hours after receiving the formal acceptance. Your manuscript will remain under strict press embargo until 2 pm Eastern Time on the date of publication. For more information, please contact globalpubhealth@plos.org.

Thank you again for supporting Open Access publishing; we are looking forward to publishing your work in PLOS Global Public Health.

Best regards,

Gauri Desai, PhD

Academic Editor

PLOS Global Public Health

***********************************************************

Reviewer Comments (if any, and for reference):
